# TriTOX: A novel *Trichomonas vaginalis* assay platform for high-throughput screening of compound libraries

**DOI:** 10.1016/j.ijpddr.2021.01.001

**Published:** 2021-02-10

**Authors:** Alexander Y.F. Lam, Daniel Vuong, Aaron R. Jex, Andrew M. Piggott, Ernest Lacey, Samantha J. Emery-Corbin

**Affiliations:** a Population Health and Immunity Division, The Walter and Eliza Hall Institute of Medical Research, Melbourne, VIC, Australia; b Microbial Screening Technologies, Smithfield, NSW, Australia; c Department of Veterinary Biosciences, Melbourne Veterinary School, Faculty of Veterinary and Agricultural Sciences, The University of Melbourne, Parkville, VIC, Australia; d Department of Molecular Sciences, Faculty of Science and Engineering, Macquarie University, North Ryde, NSW, Australia; e Department of Medical Biology, The University of Melbourne, Parkville, VIC, Australia

**Keywords:** *Trichomonas*, *Tritrichomonas*, Drug-discovery, Natural products, Microbial metabolites, MIC, Minimum inhibitory concentration, NI, Nitroimidazole, STD, Sexually-transmitted disease, HIV, Human immunodeficiency virus, Mtz, Metronidazole, DMSO, Dimethyl sulfoxide, TSA, Trichostatin A, PBS, Phosphate buffered saline, MetAP2, Methionine aminopeptidase 2, BLAST, Basic local alignment search tool, ADT, AutoDockTools, I-TASSER, Iterative threading assembly refinement algorithm, PDB, Protein data bank, MOA, Mode of action, MTS, Medium-throughput screen, HTS, High-throughput screen

## Abstract

*Trichomonas vaginalis* is a neglected urogenital parasitic protist that causes 170 million cases of trichomoniasis annually, making it the most prevalent non-viral, sexually transmitted disease. Trichomoniasis treatment relies on nitroheterocyclics, such as metronidazole. However, with increasing drug-resistance, there is an urgent need for novel anti-trichomonals. Little progress has been made to translate anti-trichomonal research into commercialised therapeutics, and the absence of a standardised compound-screening platform is the immediate stumbling block for drug-discovery. Herein, we describe a simple, cost-effective growth assay for *T. vaginalis* and the related *Tritrichomonas foetus*. Tracking changes in pH were a valid indicator of trichomonad growth (*T. vaginalis* and *T. foetus*), allowing development of a miniaturised, chromogenic growth assay based on the phenol red indicator in 96- and 384-well microtiter plate formats. The outputs of this assay can be quantitatively and qualitatively assessed, with consistent dynamic ranges based on Z′ values of 0.741 and 0.870 across medium- and high-throughput formats, respectively. We applied this high-throughput format within the largest pure-compound microbial metabolite screen (812 compounds) for *T. vaginalis* and identified 43 hit compounds. We compared these identified compounds to mammalian cell lines, and highlighted extensive overlaps between anti-trichomonal and anti-tumour activity. Lastly, observing nanomolar inhibition of *T. vaginalis* by fumagillin, and noting this compound has reported activity in other protists, we performed *in silico* analyses of the interaction of fumagillin with its molecular target methionine aminopeptidase 2 for *T. vaginalis*, *Giardia lamblia* and *Entamoeba histolytica*, highlighting potential for fumagillin as a broad-spectrum anti-protistal against microaerophilic protists. Together, this new platform will accelerate drug-discovery efforts, underpin drug-resistance screening in trichomonads, and contributing to a growing body of evidence highlighting the potential of microbial natural products as novel anti-protistals.

## Graphical abstract

**Figure:**
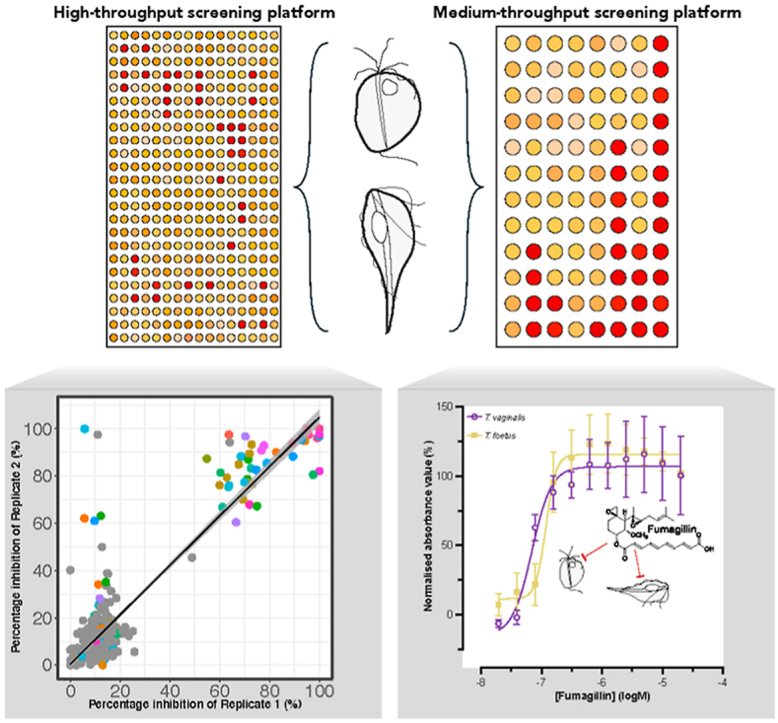

## 1 Introduction

*Trichomonas vaginalis* is a microaerophilic parasitic protist that infects the human urogenital tract causing trichomoniasis, the most prevalent non-viral sexually-transmitted disease (STD) worldwide. It is estimated ~170 million cases of trichomoniasis occur globally, of which 85% are in developing countries (Harp and Chowdhury, 2011; Newman et al., 2015). However, these rates are likely to vastly underestimate true prevalence, given the high rates of infections lacking acute symptomology (i.e., ‘asymptomatic’), and that trichomoniasis is often nonreportable even in developed countries, with diagnostic resources reliant on other reproductive or STD surveillance and testing (Lusk et al., 2010; Regan et al., 2020). Symptomatic *T. vaginalis* infections occur primarily in women and cause vaginitis, urethritis and cervicitis, while in men symptomatic infections are rarer, but cause urethritis and prostatitis (Secor, 2012). Although *T. vaginalis* was previously downplayed as self-clearing ‘nuisance’ infections (Hoots et al., 2013; Kissinger, 2015; Van der Pol, 2007), public health concerns are increasing due to significant primary diseases burdens and reproductive tract sequalae, including pelvic inflammatory disease, adverse pregnancy outcomes (pre-term birth/miscarriage, low birth weight), infertility and cervical cancer (Cotch et al., 1997; Kissinger, 2015; Klebanoff et al., 2001; Van der Pol, 2007). Moreover, *T. vaginalis* infections produce advantageous urogenital environments for other STDs, including strong associated with increased viral loads and transmission of Human Immunodeficiency Virus (HIV) (Buve et al., 2001; Kissinger, 2015; Sorvillo and Kerndt, 1998), and these coinfections are correlated with worse clinical outcomes than singular infections (Nolan et al., 2020; Van der Pol, 2007).

Current chemotherapeutics for trichomoniasis are limited to the 2-methyl-5-nitroimidazole (NI) drugs, metronidazole (Mtz) and tinidazole (Bouchemal et al., 2017; Kissinger, 2015; Secor, 2012). To date, a single oral dose of Mtz remains the recommended and most clinically efficacious treatment, however rates of recurrent infection in women are 5–30%, but also as high as 37% in HIV-infection populations (Howe and Kissinger, 2017). For these reoccurring or single-dose refractory infections, increased dose and/or multi-dose regimes are prescribed, including a switch to tinidazole, which often leads to clearance (Cudmore et al., 2004). However, as recurrent infections are often refractory due to drug resistance rather than reinfection (Crowell et al., 2003; Kirkcaldy et al., 2012; Kissinger et al., 2008), concerns have arisen that increased dose regimes may select for higher levels of Mtz resistance and NI cross-resistance (Crowell et al., 2003; Cudmore et al., 2004; Kirkcaldy et al., 2012; Mammen-Tobin and Wilson, 2005; Schwebke and Barrientes, 2006). Furthermore, increased doses are associated with increased side-effects, which are particularly common for Mtz (Crowell et al., 2003). Unfortunately, where contraindications or repeatedly refractory infections occur, the few alternative treatments are usually intravaginal and less effective than oral, systemic treatments (Secor, 2012).

There are no new compounds currently in clinical trials for *T. vaginalis.* Given the global prevalence of trichomoniasis, the paucity of current and alternative treatment options, and the increasing emergence of drug resistance, there is an urgent need to expedite drug-screening and drug-discovery. A lack of standardised screening methods have stymied drug-screening efforts, with many studies reliant on manual microscopy-based estimates of minimum inhibitory concentration (MIC) ranges, while others have ranged from either metabolically activated dyes or radioactive labelling of parasites (Crowell et al., 2003; Duarte et al., 2009; Goodhew and Secor, 2013; Upcroft and Upcroft, 2001). Recently an image-based high-content screening platform was developed (King et al., 2019), however this uses technological platforms which are not yet widely available, and required additional parasite labelling and handling. Overall, there remains a need for a simple and cost-effective screening platform for *T. vaginalis* without requirements for expensive platforms or reagents and which can be used as a standard approach across laboratories. In particular, the proposed assay should be capable of automation for high-throughput screens, and to able to precisely calculate IC_50_, as this is of added value for routine drug-resistance screening.

Herein we describe a simple, chromogenic *T. vaginalis* trophozoite growth assay, which also is amenable for the distantly related *Tritrichomonas foetus*, a significant veterinary pathogen causing analogous sexually-transmitted disease in cattle (Frey and Muller, 2012), and diarrheal disease in domestic cats (Yao and Koster, 2015). As in *T. vaginalis*, *T. foetus* has been underserved in its drug-discovery based on insufficient commercial incentives, leaving no approved treatment in the USA where metronidazole is not licensed for food-animal use (Bendesky et al., 2002). Our assay tracks trichomonad growth via absorbance and was able to (1) identify anti-trichomonal compounds that killed parasites or inhibited growth, (2) be miniaturised for both medium- and high-throughput microtiter formats (3) applied in high-content, single concentration screens as well as dilution series for plotting precise and reproducible dose-response curves. We have demonstrated the value of this assay platform for discovering new anti-trichomonal and anti-protistal compounds through screening a new microbial natural product library of >800 pure compounds. Microbial natural products have demonstrated and significant successes as anti-protistals, particularly noting the antibiotic azomycin was the first nitroimidazole and the chemical precedent for synthesis of the widely used and essential 5-nitroimidazole compounds (Nakamura, 1955), trichostatin A was the first HDAC inhibitor and progenitor of the hydroxamate histone deacetylase inhibitors (Vanhaecke et al., 2004), while staurosporine was the first pan-active protein kinase C inhibitor all derived from *Streptomyces* spp. (Tamaoki et al., 1986). This screening data contributes to increasing evidence driving renewed interest in microbial natural products as anti-trichomonals (Gehrig and Efferth, 2009; King et al., 2019), as well as potential mechanisms and scaffolds that could be targeted for further exploration, identifying pivotal sites of action in eukaryotes that are fundamental to modern anti-tumour discovery.

## 2 Materials and methods

### 2.1 Parasite lines and *in vitro* culture

The *Trichomonas vaginalis* G3 isolate (ATCC PRA-98, ‘Donne’) was a gift from Jacqui Upcroft and Peter Upcroft while the *Tritrichomonas foetus* Kv1 isolate (ATCC 30924, ‘Riedmuller) was a gift from Jan Slapeta. Both species were maintained in TYI-S-33 media (Clark and Diamond, 2002) supplemented with 10% bovine serum at 37 °C in flat-sided 10 mL tubes (Nunclon delta) filled and capped tightly, with subculturing performed every 36–48 h.

### 2.2 Compounds and microbial metabolite library

Metronidazole (Mtz, Sigma Aldrich) was used from a 100 mM stock dissolved in dimethyl sulfoxide (DMSO), trichostatin A (TSA) and staurosporine (BioAustralis, Sydney) were 1 mM stocks. The ‘BioAustralis Discovery Plates’ compounds produced by BioAustralis were provided for drug-screening, and featured a new microbial natural metabolite library of 812 pure compounds. A full list of microbial metabolites in the library can be found in Supplementary Data 1. Compounds in the BioAustralis Discovery Plates included purified microbial metabolites stocks dissolved at 1 mg/mL in DMSO, which were spotted into 384-well plates (Greiner Bio-One, #781098) at 25 nL and stored at −80 °C. Compounds used in confirmatory medium-throughput screens (MTS) were provided by BioAustralis (Smithfield, Sydney) and included tubercidin, toyocamycin, toxoflavin, sparsomycin, L 156602, leptomycin A, leptomycin B, kazusamycin A, penicolinate A, TSA, trichostatin C, apicidin, emestrin, fumagillin, gliotoxin, putative gliotoxin analogue (molecular weight: 260) and azomycin. All compounds were dissolved to 10 mM stocks in DMSO and stored at −20 °C.

### 2.3 Establishing and optimising chromogenic assay conditions

For microtiter plate assays, trichomonads were grown in anaerobic conditions within GasPak EZ anaerobe pouches with fresh anaerobic sachets. Working reagent of the chromogen was phenol red (Sigma) in a 1% w/v working solution in phosphate buffered saline (PBS), which was filter sterilised. Chromogen absorbance was monitored at 570 nm on an absorbance microplate reader after plates were allowed to equilibrate in atmospheric conditions at room temperature for at least 5 min.

After absorbances were measured, data were processed as followed unless indicated otherwise in the text. For calculating dose-response curves, the baseline for 0% inhibition (DMSO-only, ‘uninhibited’) were subtracted from the entire plate and these data were then transformed to a proportional value of the ‘kill’ wells (100% inhibition) for each plate. Prism 8 (GraphPad) was used to plot dose-response curves and calculate IC_50_ values, using a least squares regression, log[inhibitor] vs normalised response (variable slope, four parameters) model. Where replicate plates were used, data for dose-response curves were plotted as the mean±SD of these replicates.

The Z′ (Equation 1) were used to quantify the suitability of assay conditions (Zhang et al., 1999). Where σ and μ represent the standard deviations and the means of both the positive and negative control (denoted by subscripts “p” and “n”) absorbance values respectively. 0.5 < Z′ ≤ 1 classified as an excellent assay; 0 < Z′ ≤ 0.5 classified as a marginal assay; Z′ ≤ 0 classified as an unsuitable assay.

$$Z^{\prime }=1-\frac{3\left(\sigma_{p}+\sigma_{n}\right)}{\left|\mu_{p}-\mu_{n}\right|}$$

Equation 1: Z′ calculation determining the suitability of assay conditions.

#### 2.3.1 Comparing *T. vaginalis* and *T. foetus* cell counts to changes in chromogen and pH

To assess correlation between protist growth and chromogen, trichomonad cell numbers, pH and absorbance were monitored for both *T. foetus* and *T. vaginalis*. Trichomonad cells were seeded at 10,000 cells/mL in 6-well plates using both 5% and 10% (v/v) chromogen concentrations in well volumes of 5 mL. Duplicate wells were used to measure pH and cell counts taken at 24, 48 and 72 h in both chromogen concentrations. Negative control wells containing media and chromogen only were measured for pH and absorbance at each timepoint, also compared to baseline absorbance, pH of media and chromogen measured at 0 h. In order to take absorbance measurements, 50 μL and 100 μL aliquots were transferred in triplicate from 6-well plates into 96-well microtiter plates (Thermo Fisher Scientific, Australia).

The effect of varying cell seeds on chromogen absorbance was further monitored for both *T. vaginalis* and *T. foetus*. In 96-well microtiter plates, trichomonad cell numbers of 20, 200 and 1000 were seeded into both 50 μL and 100 μL volumes containing either 5% or 10% (v/v) chromogen in triplicate wells. Negative control wells without cells were included for each well volume and chromogen concentration. Chromogen absorbance was measured at 4, 8, 16, 20, 27, 44, 48 and 72 h and compared to baseline absorbances measured at 0 h.

#### 2.3.2 Assessing *T. vaginalis* and *T. foetus* growth in pre-optimisation medium-throughput conditions

Trichomonad growth assays were assessed via Mtz drug-sensitivity testing in a medium throughput format (96-wells). Mtz was screened as two-fold, 10-point dilutions between 100 and 0.2 μM. For both *T. foetus* and *T. vaginalis* conditions were 5% (v/v) chromogen, well volumes of 100 μL and 200 μL and seeds of 10,000 and 20,000 cells/mL, and absorbance measured at 40 and 65 h. Mtz dose-response curves were plotted and Mtz IC_50_ calculated for both species, as well as the Z′ for each combination of conditions (volume, seed, timepoint) based on 4 technical replicates as described in Section 2.3.

#### 2.3.3 Assessing *T. vaginalis* growth in miniaturised medium- and high-throughput conditions

Miniaturised assay formats were assessed via Mtz drug-sensitivity for *T. vaginalis* in 96-well and 384-well plate formats using 5% (v/v) chromogen. For medium-throughput formats, seeds of 15,000 and 50,000 cells/mL were used in well volumes of 50 μL and 100 μL. For high-throughput formats, 25 μL well volumes were evaluated using seeds of 10,000, 20,000 and 40,000 cells/mL, while 50 μL well volumes used seeds of 5000, 10,000 and 20,000 cells/mL. Absorbance was measured at 24, 36 and 48 h, with Mtz dose-response curves, Mtz IC_50_ and the Z′ evaluated for each combination of conditions (volume, seed, timepoint) in 96- and 384-well format based on three technical replicates as described in Section 2.3.

To further evaluate whether increased chromogen was required for miniaturised (25 μL) 384-well volumes, 5% and 10% chromogen was tested in optimised conditions for *T. vaginalis* (10,000 cells/mL, 25 μL, 48 h) and used to assess drug sensitivity to Mtz, staurosporine and TSA.

### 2.4 Drug-screening of a novel microbial metabolite library in *T. vaginalis*

A compound library containing 812 purified microbial metabolites was screened against *T. vaginalis* in biological duplicates. Assay conditions were 10,000 cells/mL in well volumes of 25 μL, with absorbance measured at 48 h. Data were normalised against the minimum and maximum absorbance reading for each plate, and the % growth calculated for each compound. Compound concentrations for each well were calculated based on compound MW (Supplementary Data) and 1 mg/mL stock solutions. Compounds were considered a ‘hit’ if they displayed >50% inhibition in biological duplicates. Data visualization of inhibitory activity of compounds were done with the tidyverse (Wickham et al., 2019), ggplot2 (Wickham, 2016) and ggforce packages on RStudio.

#### 2.4.1 Comparison of compound activities of *T. vaginalis* to mammalian cell lines

Anti-tumour and cytotoxicity data for the microbial metabolite library were based on screens against mouse myeloma line NS-1 (ATCC TIB-18) and a nontumorigenic neonatal foreskin fibroblast (NFF) cell line, respectively. Inhibitory activities and compound concentrations were calculated as above and compounds considered active if they displayed >25% inhibition in either cell line. Overlap and intersection between anti-tumour, cytotoxicity and anti-trichomonal compounds were visualised using an UpSet plot (Conway et al., 2017) (https://github.com/hms-dbmi/UpSetR).

#### 2.4.2 Medium-throughput screens for hit compounds against *T. vaginalis* and *T. foetus* growth

A selection of 17 compounds assayed in the high-throughput screen (HTS) for *T. vaginalis* which were available through BioAustralis were obtained for validation of inhibitory in/activity in our optimised medium-throughput format. For this, a 10-point serial dilution was used for drug-susceptibility testing to calculate IC_50_ values of ‘hit’ compounds. Mtz was used as a positive control. Each screen was performed with 3 technical replicates and a minimum of 2 biological replicates in both *T. vaginalis* and *T. foetus* using assay conditions of seeds of 10,000 cells/mL in 50 μL, with absorbance measured at 48 h. Dose response curves and IC_50_ were calculated for each compound as described above in Section 2.3.

#### 2.4.3 Evaluation of antibacterial activity of fumagillin

Antibacterial activity of fumagillin against *Staphylococcus aureus* (ATCC 25923) and *Bacillus subtilis* (ATCC 6633) was evaluated and provided by Microbial Screening Technologies at 5 μg/mL using assay platforms (Gilchrist et al., 2020)

### 2.5 Molecular modelling of fumagillin with protistal methionine aminopeptidase orthologs

Hypothetical protein sequences of protistal methionine aminopeptidase 2 (MetAP2) were identified through Basic Local Alignment Search Tool (BLAST) on orthomcl.org, with gene accessions of protistal orthologs provided in Table 1. For molecular modelling, the ligand file for the fumagillin compound was downloaded from PubChem (Kim et al., 2019) (PubChem CID: 6917655). MetAP2 homology modelling utilised I-TASSER (Roy et al., 2010; Yang et al., 2015; Zhang, 2008) for 3D structural homology modelling for *T. vaginalis* and *Entamoeba histolytica*, with hypothetical protein structures for *Giardia lamblia* downloaded from predictein.org based on previous I-TASSER proteome modelling (Ansell et al., 2019). Binding residues on MetAP2 were identified through a literature search (Griffith et al., 1998; Kulakova et al., 2014; Liu et al., 1998), alignment of protein sequences and superimposing orthologous protein structures with crystal structures of *Homo sapiens* MetAP2 (PDB:1BOA) through UCSF Chimera (version 1.1.4)(Pettersen et al., 2004). AutoDockTools-v.1.5.6 (ADT) was used to process protein and ligand files, adding polar hydrogens, partial charges, creating flexible residues, detecting a root atom and selecting rotatable bonds for ligands. Processed fumagillin was docked with active binding residues on orthologous MetAP2 (Table 1) through AutoDock Vina (version 1.1.2)(Trott and Olson, 2010). A flexible residues parameter was used in all dockings, whereby the residues lining the binding site were made flexible to compensate for conformational selection/induction changes to the receptor prior/after ligand binding (Paul and Weikl, 2016). Binding affinities were calculated by Vina and interactions visualised through Chimera. Multiple sequence alignments were completed through Chimera and visualised through Aline, colouring residue chemistries by ALSCRIPT Calcons (Bond and Schüttelkopf, 2009).

**Table 1:** **MetAP2 and identified protistal orthologs:** Gene accessions refer to identified orthologous protein sequences through BLAST of P50579 from UniProtKB, where protistal gene accessions where retrieved from the EuPathDB databases TrichDB, GiardiaDB and AmoebaDB.

Ligands	Molecular target	Orthologs			
		*Homo sapiens*	G. lamblia	T. vaginalis	E. histolytica
**Fumagillin**	**MetAP2**	PDB:1BOA (UniProtKB – P50579)	GL50803_86600	TVAG_317020 TVAG_476160 TVAG_410220 TVAG_190580	EHI_126880

### 2.6 Data accessibility

All supplementary files have also been made available via the Mendeley data repository, including the Supplementary Information, high-resolution images for Supplementary Figs. S1–8 and Supplementary Data S1 (DOI: http://dx.doi.org/10.17632/6p2pb7s583.1).

## 3 Results

### 3.1 Correlation between trichomonad cell growth and the chromogen

**Fig. 1:**
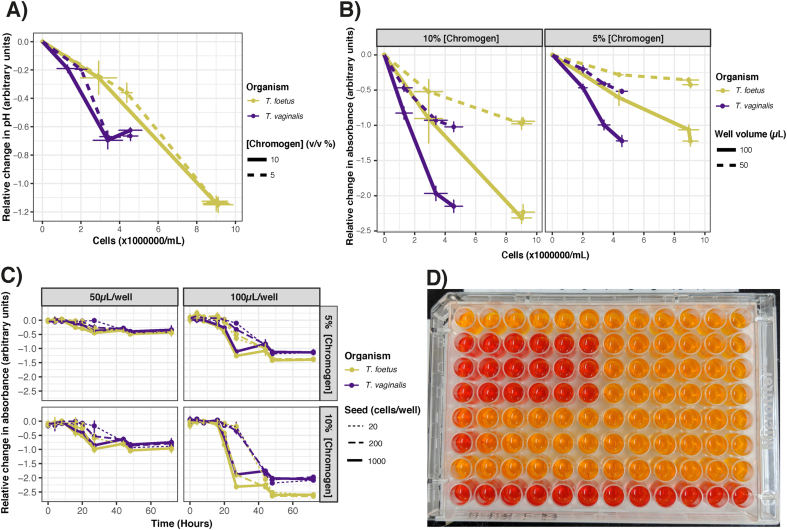
**Change in absorbance values during *T. vaginalis* and *T. foetus* growth (A).** Relative change in pH of parasite cultures compared to control during growth. Data plotted as mean ± SD with timepoints shown at 0, 24, 48 and 72 h from left to right, demonstrating cell counts increase over time. **(B).** Correlation of trophozoite numbers with change in absorbance values. Data plotted as mean ± SD with timepoints shown at 0, 24, 48 and 72 h from left to right. Data were normalised according to relative control wells (‘blank’, media-only) **(C).** Changes in absorbance values relative to control wells (‘blank’, media-only) across varying incubation conditions including variation in seed, well volume and chromogen concentration. Data plotted as mean±SD and recorded at multiple timepoints at 0, 4, 8, 16, 20, 27, 44, 48 and 72 h. **(D)**. Representative photo of *T. vaginalis* growth (10,000 cells/mL) in the presence of 5% chromogen in 200 μL wells, incubated for 40 h. Row A was supplemented with 0.5% DMSO as a vehicle control and Row H contained ‘blank’ wells (i.e. no *T. vaginalis* trophozoites). Rows B to G shows *T. vaginalis* growth in the presence of 100 μg/mL (column 1) Mtz, tinidazole, furazolidone, albendazole, quinacrine and paromomycin respectively, serially diluted 2-fold across the row.

Observations across *in vitro* cultures of *T. vaginalis* have demonstrated parasites excrete lactic and acetic acids, which causes decreases in pH of the culture media in closed conditions to as low as 4.5 (Petrin et al., 1998). Based on these observations we investigated using the pH indicator phenol red, which has a chromogenic range of yellow to red across pH 6–8, to track trichomonad growth *in vitro*. For *T. vaginalis* and *T. foetus* we confirmed that the pH of parasite cultures decreased over time as cell numbers increased during anaerobic growth in 6-well microtiter plates (Fig. 1A). This decrease in pH was greater after 24 h for *T. foetus* (~0.3) when compared with *T. vaginalis* (~0.2), and correlated with larger increases in cell growth (~4,000,000 cells/mL for *T. foetus* vs ~2,000,000 cells/mL for *T. vaginalis*). Our results supported previous observations that pH declines with trichomonad growth, with ~10-fold increased acidity as parasites entered the declining phase of cell growth at 20–30 h. In particular, almost no further changes in pH or absorbance were observed after 48 h to 72 h. This coincided with stabilised cell counts, which also did not change between these time periods (Fig. 1C). In contrast, the pH of blank media slightly declined in the first 24 h from 7.0 to 6.8, but stabilised after 24 h (Supplementary Fig. S1). Together these highlighted changes in pH could be used to assess growth of both *T. vaginalis* and *T. foetus*, and could also be quantified by measuring absorbance based on the red wavelength (570 nm) of the chromogen (Fig. 1B).

The dynamic range for absorbance could be easily adjusted by increasing chromogen or well volume. Higher chromogen concentrations and higher well volume produced a greater dynamic range of ~2.5, while inversely, lower concentrations and low well volumes had smaller dynamic ranges of ~0.5 (Fig. 1). No differences in cell count between 5% or 10% (v/v) concentrations were observed at 24, 48 and 72 h of incubation (Fig. 1), highlighting the non-toxic nature of the chromogen.

Both trichomonad species entered exponential growth phases within 24 h (Fig. 1A). Therefore, we investigated absorbance changes relative to starting cell numbers. Regardless of the initial seed number of *T. foetus* or *T. vaginalis*, absorbance values stabilised after ~44 h of anaerobic incubation (Fig. 1C), coinciding with convergence at stationary phase of growth. Together, these data support the use of quantifying pH changes as a measure of trichomonad growth, where completed exponential growth phases corresponded to the chromogenic change from red to yellow. Importantly, this was quantifiable by absorbance, but was also qualitatively appreciable by eye (Fig. 1D).

### 3.2 Pre-optimisation of assay conditions for *T. vaginalis* and *T. foetus* growth

Given demonstrated correlation between trichomonad growth and changes in the absorbance (Fig. 1), conditions were tested using Mtz-susceptibility assays to determine the optimal conditions for drug-screening. Although chemosensitivity of *T. vaginalis* to Mtz is established, the precise IC_50_ is variable. Instead, reported minimum inhibitory concentrations (MIC) *in vitro* for *T. vaginalis* ranged from 1.5 to 5 μM depending on assay type, conditions and isolates (Crowell et al., 2003; Upcroft and Upcroft, 2001), whereas *T. foetus* Mtz *in vitro* MIC values were slightly lower ranging from 1.5 to 2.5 μM (Kather et al., 2007). Assay optimisations were performed to evaluate Mtz-susceptibility by dose-response curves and calculated IC_50_ values, as well as assessment of conditions via calculation of the Z′, a statistical parameter which considers technical and systematic errors to infer assay suitability (Zhang et al., 1999). Z′ > 0.5 was considered acceptable, with scores closest to 1 considered the most optimal.

**Fig. 2:**
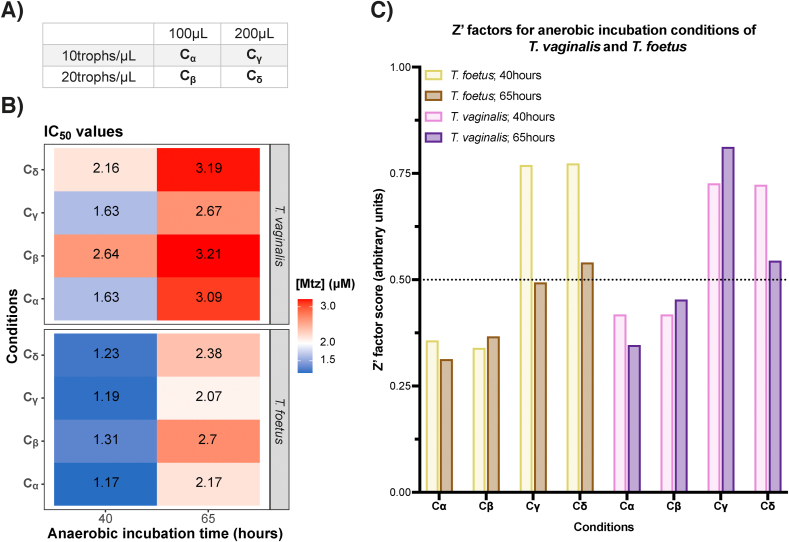
**Optimisation of assay conditions for *T. vaginalis* and *T. foetus* growth.** Assessment of several medium-throughput conditions for *T. vaginalis* and *T. foetus* growth in 96-well plates. **(A).** Anaerobic incubation conditions abbreviated based on seed and volume per well. “trophs” = trophozoites. **(B).** IC_50_ values of conditions for *T. vaginalis* and *T. foetus* incubated at 40 and 65-h. **(C).** Z′ of conditions for *T. vaginalis* and *T. foetus* incubated at 40 and 65-h. Cut-off Z′ factor of 0.5 is shown.

Mtz-susceptibility was tested in *T. foetus* and *T. vaginalis* in conditions varying seed cell numbers (low – 10,000 cells/mL; high – 20,000 cells/mL) and well volume (100 μL, 200 μL) (Fig. 2). Absorbance was measured at 40 and 65 h, at the end of log-phase and in stationary/declining phases (Fig. 1C), respectively. Although all dose response curves plotted had good sigmoidal shapes, absorbance values were more consistent at 40 h whereas large variation occurred at 65 h, particularly for *T. foetus* (Supplementary Fig. S2). Further, although dose-response curves were close to top and bottom constraints (0–100% absorbance) in *T. vaginalis* at both timepoints, *T. foetus* were considerably >100% by 65 h. This corresponds with acceptable Z′ for *T. foetus* only in conditions at 40 h, and only in higher well volumes (Fig. 2). This implicated faster growth of *T. foetus* compared to *T. vaginalis* (Fig. 1), influenced suitability of its assay conditions, and overgrowth correlated negatively with Z′. Despite this, Mtz IC_50_ values for *T. foetus* remained consistent between conditions at the same timepoint, calculated at 1.2 μM at 40 h and 2.3 ± 0.1 μM at 65 h. Although both IC_50_ are within previously reported ranges (Kather et al., 2007), given log-phase growth of *T.foetus* is completed between 36 and 48 h irrespective of cell number (Fig. 1C), absorbance for calculation of dose-response curves should be measured no later than 48 h. Further, *T. foetus* optimal assay conditions is recommended with lower cell numbers, particularly in miniaturised formats.

For *T. vaginalis*, all assay conditions with larger well volumes (200 μL) had Z′ > 0.5 at both timepoints. As observed for *T. foetus*, IC_50_ values were higher at 65 h 3.0 ± 0.1 μM compared to 40 h 2.0 ± 0.2 μM, but were more variable at the earlier timepoint. Both measurements were within range of the previously reported MIC for Mtz (Upcroft and Upcroft, 2001). Therefore, we decided further optimisation of assay conditions was required for *T. vaginalis*, specifically from the view to miniaturise assay well volumes in medium- and, particularly, for high-throughput drug-screening.

### 3.3 Miniaturisation of assay conditions for *T. vaginalis* growth

**Fig. 3:**
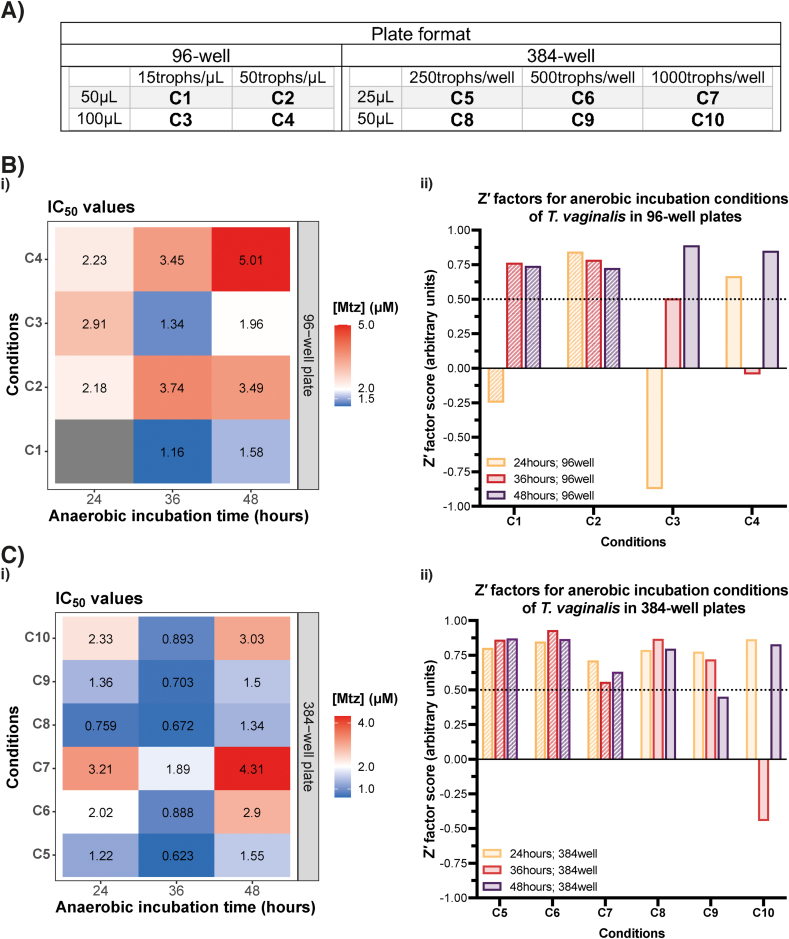
**Miniaturised assay conditions for *T. vaginalis.* (A).** Anaerobic incubation conditions tested and their abbreviation based on seed and volume per well in 96- and 384-well plates. “trophs” = trophozoites. **(B).**i). IC_50_ values of conditions for *T. vaginalis* recorded at 24, 36 and 48 h timepoints in 96-well plates. ii). Z′ factors of conditions for *T. vaginalis* recorded at 24, 36 and 48 h timepoints in 96-well plates. The Z′ factor cut-off of 0.5 is shown as a dotted line. **(C).**i). IC_50_ values of conditions for *T. vaginalis* recorded at 24, 36 and 48 h timepoints in 384-well plates. ii). Z′ factors of conditions for *T. vaginalis* recorded at 24, 36 and 48 h timepoints in 384-well plates. Cut-off Z′ factor of 0.5 is shown as a dotted line.

Further optimisation of *T. vaginalis* assay conditions focused on reduced well volumes, and the selection of the optimal timepoint for assay measurement and conditions. As above, Mtz-susceptibility was used to generate dose response curves, IC_50_ and Z′ for varying well volumes and cell seeds (Fig. 3, Supplementary Fig. S3). Given timepoints in declining phases of cell growth were more variable (Fig. 2), timepoints during (24 h) and after (36, 48 h) exponential or log-phase growth were evaluated. While Z′ were consistently above Z′ cut-offs for all conditions when measured at 48 h, particularly in 96-well formats, dose response curves were sigmoidal at 48 h for 384-well plates (Supplementary Fig. S3). Mtz IC_50_ values did not cluster by timepoint in contrast to preoptimization experiments (Fig. 2B), but were influenced by assay conditions (well volume, seed cell numbers). Therefore, we selected conditions that were optimal for lower well volumes, with medium-throughput conditions of 50 μL well volume and 15,000 cells/mL seed, and high-throughput conditions of 25 μL well volume and 10,000 cells/mL seed. We selected an assay endpoint of 48 h for both selected assays formats, noting Mtz IC_50_ were near equivalent (~1.5 μM) when read at this timepoint.

Chromogen experiments demonstrated absorbance values were higher when well volumes and chromogen concentrations were increased (Fig. 1). As optimisation of assay conditions (Figs. 2 and 3) were performed using 5% (v/v) chromogen, further investigations into optimal chromogen concentrations were performed given priority for miniaturised well volumes. The effect of chromogen concentrations of 5 and 10% (v/v) were evaluated in dose-response profiles of three reported anti-protistal compounds, Mtz, TSA and staurosporine. Both chromogen concentrations produced near-identical dose-response curves and IC_50_ values when fitted using the same nonlinear, least squares regression model (Supplementary Fig. S4A). Further, both concentrations had a suitable Z′ of 0.680 and 0.749 for 5% and 10% (v/v) chromogen respectively. However, the absolute difference between positive and negative absorbance values were twice as high when using 10% (v/v) chromogen (Supplementary Fig. S4B), producing a higher dynamic range when higher chromogen concentrations were used also verified qualitatively (Supplementary Fig. S5). In order to compensate for lower well volumes in our miniaturised conditions, 10% (v/v) chromogen concentration was used in further drug-screening experiments.

### 3.4 Screening a microbial metabolite library against *T. vaginalis*

**Fig. 4:**
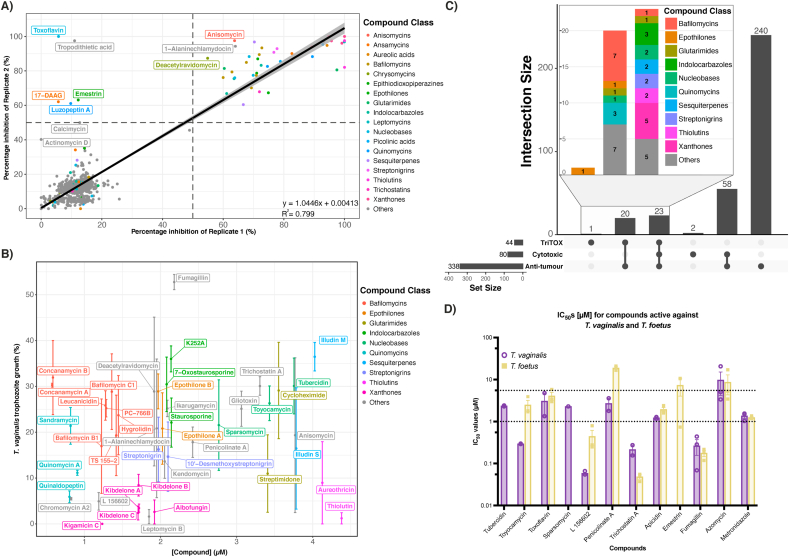
**Assessing the anti-trichomonal activity of a novel microbial metabolite library. (A).** Correlation between % inhibition of *T. vaginalis* trophozoite growth between biological duplicate screens of the microbial metabolite library. Each data point corresponds to a single compound and dashed lines indicate 50% inhibition for each replicate. Compounds are assigned a colour if at least one analogue from its structural class demonstrated >50% inhibitory activity in both biological duplicates. Individual compounds varying by > 30% in inhibitory activity between replicates are also labelled. **(B).** Scatterplot of anti-trichomonal compounds with demonstrated >50% inhibition in both biological duplicates, showing percentage growth (%) and their corresponding calculated micromolar concentrations (μM). Data are plotted as mean ± SEM, n = 2, and each data point corresponds to a single compound. Compounds are assigned a colour if at least two analogues from its structural class demonstrated >50% inhibitory activity in both biological duplicates. **(C).** IC_50_ values of anti-trichomonal compounds identified from high-throughput screening of the library for both *T. vaginalis* and *T. foetus*. Dashed line drawn at 1 and 5 μM. Data plotted as mean±SEM, biological replicates shown as individual data points over bars (n = 2 or n = 3). **(D).** UpSet plot depicting the overlap between anti-trichomonal (TV) activity of microbial metabolites with cytotoxic (NFF) and anti-tumour (NS-1) activity, where the filled circles on the x-axis indicate the intersection of activities and the height of the bars show the size of intersections. Compounds are assigned a colour if at least two analogues from its structural class demonstrated >50% inhibitory activity against *T. vaginalis* in both biological duplicates. (For interpretation of the references to color in this figure legend, the reader is referred to the Web version of this article.)

Using established conditions optimised for high-throughput (25 μL, 10,000 cells/mL seed, 10% (v/v) chromogen, 48 h), we conducted a screen against *T. vaginalis* growth using the BioAustralis Discovery Plates, a new natural product compound library. These plates consisted of 812 compounds which are predominately microbial metabolites and semi-synthetic analogues, including metabolites with well understood modes of action (MOA), and many rare metabolites with no described bio-activity profile. We used this new compound library to confirm the presence of known MOA in our bioassay as well as new actives, and explored the broader potential of anti-protistals among metabolites of microbial origins. Compounds in the BioAustralis Discovery Plates represent a diverse range of microbial metabolites, with 603/812 of these classified, based on important structural moieties, among ~130 compound classes. Based on biological duplicate screens, we identified 43 compounds (5.30% of compounds in the library) with reproducible > 50% growth inhibition (Supplementary Data 1). We also visually identified fumagillin as a hit, where the difference in colour (as compared to negative controls) was obvious to the eye and further confirmed through visual inspection under the microscope. Of these 44 anti-trichomonal hits (Supplementary Data 1), 33 of these were from 10 compound classes, including the macrocyclic lactones (8 compounds) and polycyclic xanthones (5 compounds) (Fig. 4B).

Replicate testing in the HTS (Fig. 4A) had an R^2^ value of 0.799, and a linear curve fitted between replicate data had a slope of 1.04, with only 10/812 compounds inconsistent between replicates. From these latter compounds, 5 partial hits were identified: toxoflavin, 17-dimethylaminoethylamino-17-demethoxygeldanamycin (17-DAAG), emestrin, luzopeptin A and tropodithietic acid, where > 50% inhibition was observed only in one replicate. None of these partial hits were structurally related to each other. With the exception of 17-DAAG, the metabolites all exhibit poor solubility in most organic solvents. We suspect solubility issues affected these compounds which, when dissolved in DMSO, may have formed micro-suspensions when added to our media, leading to higher localised concentrations within wells.

Although only a few compounds have been reported as anti-trichomonal *in vitro*, our assay supported the anti-trichomonal activity of anisomycin (Jimenez and Vázquez, 1979; Sears and O'Hare, 1988), fumagillin (Seneca and Ides, 1953), thiolutin (Jimenez et al., 1973), ikarugamycin (Jomon et al., 1972), TSA (Omura and Oiwa, 1980), toyocamycin (Wright et al., 2010) and staurosporine (Chose et al., 2002). We identified additional active analogues of toyocamycin, staurosporine and thiolutin (Supplementary Fig. S6). Toyocamycin, tubercidin and sparsomycin are nucleobase metabolites. *Trichomonas vaginalis* lacks de novo purine and pyrimidine synthesis pathways (Harris et al., 1988; Miller and Lindstead, 1983), hence nucleobase metabolites may out-compete and impair nucleotide scavenging pathways that are essential for *T. vaginalis* survival (Wright et al., 2010; Zang et al., 2005). Staurosporine and its active analogues K252A and 7-oxostaurosporine are indolocarbazoles, and proposed as broad protein kinase inhibitors (Akanishi et al., 1986; Fabre et al., 1994; Omura et al., 1977). Thiolutin and aureothricin are structural analogues, inhibit RNA polymerases to halt RNA synthesis in fungi (Jimenez et al., 1973; Tipper, 1973), with a similar MOA described against other eukaryotic systems.

Of the 11 compounds that were not part of a larger structural class, anisomycin had been previously reported in other screens to have broad-spectrum anti-parasitic activity in at least 3 other parasite species (Ehrenkaufer et al., 2020; Sears and O'Hare, 1988). Anisomycin is proposed to inhibit protein synthesis specifically in eukaryotic cells, blocking peptide bond formation on eukaryotic ribosomes (Jimenez and Vázquez, 1979). It has been used historically as an anti-amoebic agent, including for *E. histolytica*, and is active against Mtz-resistant *E. histolytica* at micromolar concentrations (Ehrenkaufer et al., 2018; Gonzalez, 1956).

Our assay identified a high hit-rate of activity among compound classes not previously reported as anti-trichomonals, including the macrocyclic lactones, bafilomycins, and polycyclic xanthones, kibdelones. In particular 8/10 bafilomycin related metabolites and 5/5 polycyclic xanthones analogues were identified as active in our HTS and constituted the most active anti-trichomonals from a compound class perspective (Supplementary Fig. S6). Bafilomycins have been described as specific, potent inhibitors of Vacuolar-type H^+^-ATPase, forming relatively conserved covalent interactions with the c-subunit across both prokaryotic and eukaryotic cells (Bowman et al., 1988; Dröse and Altendorf, 1997). However, despite demonstrating promising biological activity as anti-tumour agents (Ratnayake et al., 2007), polycyclic xanthones have limited attention and their molecular target(s) are as yet unresolved.

### 3.5 Comparison of anti-trichomonal to cytotoxity and anti-tumour activity

After assessing their anti-trichomonal profile in HTS, we explored the selectivity of these compounds to assist with determining which had greatest potential to be developed into novel anti-trichomonals, as well as exploring the overlap of shared MOA between protistal and mammalian cell lines. We obtained compound activities from the same metabolite library against mammalian cell lines NS-1 (myeloma) and NFF (fibroblasts) screened in single concentration formats, which were used to classify anti-tumour and cytotoxic activity, respectively. Of the 44 anti-trichomonal hits, only epothilone B was selective for *T. vaginalis* (Fig. 4C). These microtubule-stabilising agents (Bollag et al., 1995) have not been reported to be anti-trichomonal in the literature, but have been previously reported as anti-giardial (Chen et al., 2011). Epothilones have been studied within anti-tumour contexts as anti-mitotic agents, specifically against drug-resistant tumour cells (de Jonge and Verweij, 2005; Goodin et al., 2004).

Aside from epothilones, glutarimides, bafilomycins and the nucleobases, the anti-trichomonal compounds were either anti-tumour only, or both anti-tumour and cytotoxic (Fig. 4C), potentially indicating of the conserved MOA across eukaryotes within structural classes of some of these metabolites. For example, the quinomycins have been described as bis-intercalators of DNA (Waring and Wakelin, 1974), which occurs at the minor groove of DNA through the insertion of two planar, heteroaromatic rings around two base pairs (Rackham et al., 2013). These ligand-DNA interactions have been described in context of anti-tumour activity as selective towards rapid dividing cells, causing inhibition of DNA replication and transcription, perturbating the cell cycle and interfering with DNA repair mechanisms (Nichol et al., 2019; Rahman et al., 2019). Further, anti-protistal activity of the quinomycins through targeting rapid dividing cells have been demonstrated in *E. histolytica* (Espinosa et al., 2012) and *Plasmodium falciparum* (Lee and Inselburg, 1993). We hypothesise that metabolic and DNA replicative demands of *T. vaginalis* may be similar to tumour cell lines, which correlated with the overlaps as observed between anti-trichomonal and anti-tumour activity.

### 3.6 Further assessment of HTS hits through medium-throughput assays

Next, we explored the assay's reliability and reproducibility in medium-throughput formats for drug-susceptibility testing. We assessed its performance for a selection of pure metabolites from the BioAustralis Discovery Plates. These encompassed a more diverse range of compound chemistries and in/active compounds based on the HTS. We assessed 9 active, 2 partial and 6 inactive compounds in MTS for *T. vaginalis* and *T. foetus* across a 10-point dilution series. These compounds included available structural analogues for the nucleobase, leptomycin, trichostatin and gliotoxin compound classes, which all had at least one active analogue identified in the HTS (Fig. 4A), as well as 6 structurally unrelated metabolites. We confirmed the HTS results for 10/16 compounds (7/9 true positives and 3/5 true negatives, with one partial hit confirmed inactive and the other partial hit confirmed active) in *T. vaginalis* and successfully plotted dose-response curves and calculated IC_50_ values for a range of structural chemistries (Fig. 4D, Supplementary Fig. S7).

Tubercidin, toyocamycin, sparsomycin, L 156602, penicolinate A, TSA and fumagillin were confirmed as true positives, and leptomycin A, kazusamycin A and trichostatin C were true negatives. Leptomycin B and gliotoxin (and its putative analogue not in the HTS) showed no activity in the MTS, even at the highest dose tested (~6-fold higher than in the HTS). Toxoflavin, azomycin and apicidin (partially active in the HTS) were confirmed active in the MTS. Lastly, emestrin remained inconsistent between biological replicates in our MTS against *T. foetus* but was inactive against *T. vaginalis* (Table 2, Fig. 4D). This coincided with its inconsistent behaviour in the HTS and may point to issues with compound solubility.

**Table 2:** **Compounds and their respective IC**_**50**_**values against *T. vaginalis* and *T. foetus*:** Micromolar IC_50_ values are reported as mean ± SEM. Compounds with no appreciable activity at the highest dose tested were recorded with IC_50_ > 10. “A”, “P”, “I” indicate compounds that were active, partially active and inactive in the *T. vaginalis* HTS respectively; * indicates compounds which were not present in the HTS.

Compounds	*T. vaginalis* HTS results	MTS confirmatory results	
		***T. vaginalis* IC**_**50**_**(μM ± SEM)**	***T. foetus* IC**_**50**_**(μM ± SEM)**
**Tubercidin**	A	2.4 ± 0.1	>10
**Toyocamycin**	A	0.30 ± 0.01	2.4 ± 0.7
**Sparsomycin**	A	2.3	>10
**L 156602**	A	0.059 ± 0.004	0.44 ± 0.18
**Leptomycin B**	A	>10	>10
**Penicolinate A**	A	2.7 ± 0.9	19 ± 1
**Trichostatin A**	A	0.22 ± 0.06	0.048 ± 0.006
**Fumagillin**	A	0.26 ± 0.11	0.18 ± 0.03
**Gliotoxin**	A	>10	>10
**Toxoflavin**	P	3.1 ± 1.7	4.1 ± 0.9
**Emestrin**	P	>10	7.3 ± 3.3
**Leptomycin A**	I	>10	>10
**Kazusamycin A**	I	>10	>10
**Trichostatin C**	I	>10	>10
**Apicidin**	I	1.2 ± 0.1	1.9 ± 0.2
**Azomycin**	I	9.7 ± 5.5	8.6 ± 4.3
**Gliotoxin analogue**	*	>10	>10
**Metronidazole**	*	1.4 ± 0.2	1.2 ± 0.1

Several compounds had potential species-specificity differentiating *T. vaginalis* and *T. foetus*, supporting observed genetic difference and distance between these species (Frey and Muller, 2012). The MTS platform highlighted the selective activity against *T. vaginalis* but not *T. foetus* of tubercidin, sparsomycin and penicolinate A, all of which demonstrated micromolar IC_50_ values against *T. vaginalis*, whereas only penicolinate A had weak activity against *T. foetus* (Table 2, Fig. 4D). Although tubercidin and sparsomycin was active only for *T. vaginalis*, the other nucleobase analogues toyocamycin and toxoflavin remained active against both *T. vaginalis* and *T. foetus* (Table 2, Fig. 4D).

Interestingly, azomycin (inactive in *T. vaginalis* HTS) demonstrated inconsistent activity and was among the least potent within our validatory MTS, with considerably different IC_50_ values between replicates for both protists (Fig. 4D). This is despite azomycin being the first nitroimidazole with demonstrated anti-protistal activity (Maeda, 1953). Further, we confirmed that the histone deacetylase inhibitor apicidin, though inactive in both *T. vaginalis* HTS replicates, had micromolar IC_50_ values against *T. vaginalis* and *T. foetus* (Table 2, Fig. 4D) in MTS. Lastly, we confirmed the potent activities of L 156602, TSA and fumagillin against both protists, observing sub-molar IC_50_ values against both species, but noted these potencies varied between species (Table 2, Fig. 4D).

### 3.7 Fumagillin is an inhibitor of microaerophilic protists

Fumagillin had near nanomolar potency against both protists (Table 2, Fig. 4D), and is particularly interesting as this compound and its structural analogues have extensive anti-parasitic activity, including anti-amoebic, anti-giardial and anti-plasmodial effects (Chen et al., 2009; Killough et al., 1952; Kulakova et al., 2014). In addition to demonstrating wider anti-protistal activity in *T. vaginalis*, fumagillin demonstrated no anti-bacterial nor cytotoxic activity in this study, suggesting it could be developed as a selective, potent and broad-spectrum anti-protistal compound. Fumagillin and epoxide analogues specifically inhibit methionine aminopeptidase 2 (MetAP2) (Arico-Muendel et al., 2009b; Chen et al., 2009; Griffith et al., 1998; Liu et al., 1998), which catalyses N-terminal methionine excision (NME) during co-translational protein maturation ensuring correct protein function, making it an essential gene in all organisms, although significant sequence differences are observed between human and protist orthologs (Zhang et al., 2002).

**Fig. 5:**
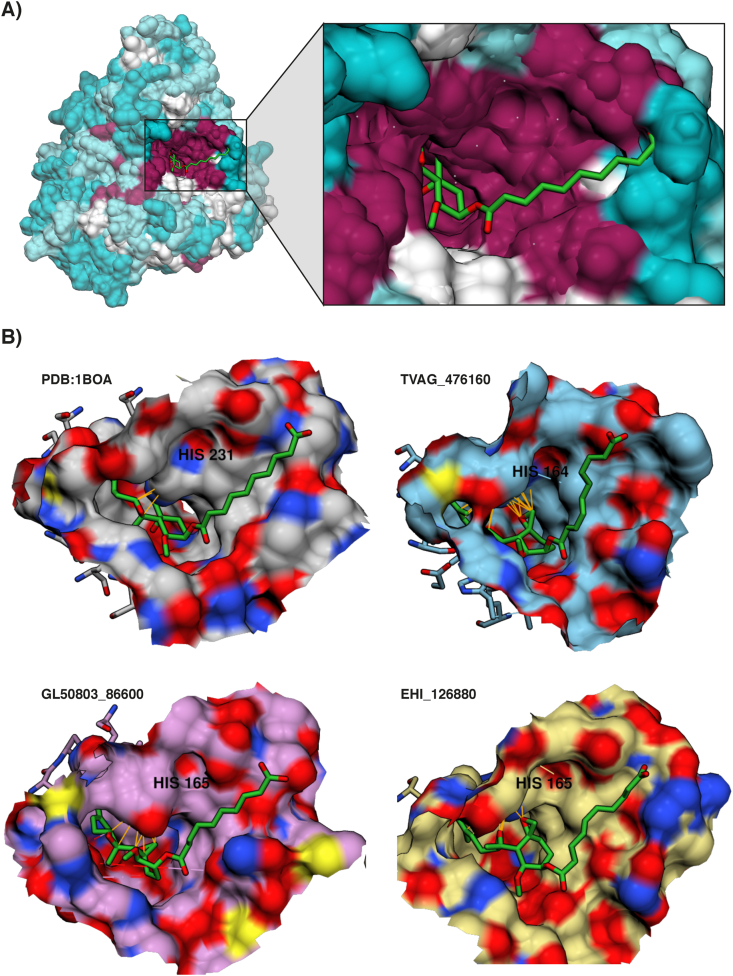
**Conserved molecular interactions between protistal MetAP2 and fumagillin. (A)**. Superimposition of human and protistal MetAP2 demonstrated the conserved fumagillin binding site. Orientation of the fumagillin molecule is identical in its binding orientation on human MetAP2 as shown in PDB:1BOA. Conservation of residues are depicted through colour as following: maroon represents 100% conservation between orthologs, white represents 75% conservation between orthologs, cyan represents <50% conservation between orthologs. **(B)**. Molecular models of fumagillin and respective MetAP2 orthologs. Models are designated labels on the top left of each model. Structures are listed from left to right as follows: PDB:1BOA: Crystal structure of fumagillin in complex with human MetAP2; TVAG_476160: fumagillin docked with a representative *T. vaginalis* MetAP2 ortholog; GL50803_86600; EHI_126880: fumagillin docked with *G. lamblia* and *E. histolytica* MetAP2 ortholog. Models are coloured by heteroatoms where red = oxygen atoms, blue = nitrogen atoms and yellow = sulfur atoms. Van Der Waals interactions between fumagillin and important histidine residue are labelled in orange lines. Histidine residue and their positions are labelled in black within each model. (For interpretation of the references to color in this figure legend, the reader is referred to the Web version of this article.)

To assess conservation of the MOA of fumagillin we used published structural-activity relationship data (Griffith et al., 1997, 1998; Han et al., 2000) and undertook *in silico* prediction of its interaction with protistal MetAP2. First, we modelled and observed the conserved fumagillin binding site in *T. vaginalis*, *G. lamblia* and *E. histolytica* as compared to experimentally solved crystal structures of human MetAP2 (PDB:1BOA (Liu et al., 1998):). This modelling predicted near 100% conservation of the active site of human MetAP2 and orthologs from *T. vaginalis*, *G. lamblia* and *E. histolytica* (TVAG_476160, GL50803_86600 and EHI_126880; Fig. 5A, Supplementary Fig. S8), including conservation of a histidine residue at position 231, which has been strongly implicated in fumagillin binding of the human ortholog (Supplementary Fig. S8). *In silico* docking based on these protistal protein models predicted molecular interactions consistent with the reported protein-ligand binding of fumagillin with MetAP2 (Griffith et al., 1998). We calculated the binding affinity of fumagillin at −6.6 kcal/mol, −7.6 kcal/mol and −6.5 kcal/mol with *T. vaginalis*, *G. lamblia* and *E. histolytica* MetAP2, respectively. Due to software limitations, we could not model covalent bond formations. Such a bond is formed between histidine 231 in human MetAP2 and the ring-epoxide moiety of fumagillin (Griffith et al., 1998). As observed, this histidine is indeed conserved in all protistal MetAP2 orthologs (Fig. 5B) and given that covalent bonds are up to 10 times stronger than Van Der Waals forces (Aljoundi et al., 2020; Lodish et al., 2000), our reported binding affinities are likely underestimates.

## 4 Discussion

Although NI drugs remain largely efficacious, drug-resistant *T. vaginalis* is increasing in frequency and resistance levels (Goodhew and Secor, 2013; Secor, 2012). Now is the window of opportunity to significantly increase effort to discover new anti-trichomonal compounds, particularly outside the NI class, before difficult-to-treat infections become completely refractory. As the majority of trichomoniasis infections occur in lower socioeconomic populations, underinvestment in diagnostic and discovery platforms has created a deficit in upcoming effective oral treatments in clinical trials. Herein, we have provided a chromogenic assay (‘TriTOX’) which can be used to rapidly and inexpensively screen thousands of compounds, and therefore accelerate drug-discovery efforts in this neglected protist. In this study we have demonstrated miniaturisation to 384-well plates, but further miniaturisation (e.g. scaling to 1536-well microtiter plates) and automation should be possible with minimal additional optimisation.

TriTOX is a simple, cost-effective and consistent *in vitro* trophozoite growth assay for *T. vaginalis* that is compatible with medium- and high-throughput screening. We have highlighted its applicability in high-content screens of large compound libraries of interest, making it an important new tool for discovery, noting its consistent Mtz IC_50_ values and dose-response profiles could be applied for standardised large-scale screens for Mtz-resistant *T. vaginalis*, replacing MIC estimates. This chromogenic assay requires no expensive reagents or advanced technological platforms, making it suitable within many lab settings as opposed to specialised methods. Moreover, no additional staining or handling of parasites are required, and the chromogenic shift can be measured quantitatively and qualitatively, if necessary. In optimised and miniaturised assay conditions, we achieved a suitable dynamic range and consistency between inter-week replicates, reflected by Z′ values at 0.741 and 0.870 for our MTS and HTS conditions respectively. These scores are within ranges of recent MTS (96-well) image-based assays in *T. vaginalis* (King et al., 2019) and *G. lamblia* (Hart et al., 2017). Further, we highlight this assay is flexible enough to accommodate rapid growth while retaining consistency across many conditions and ranges. As the chromogen is non-toxic, intact parasites can be combined with imaging post-quantification to confirm results and correlate any morphological changes in response to compounds. Further, this assay allows inter-species comparison of compound activities between *T. vaginalis* and *T. foetus*. By sharing the same standardised assay between these species, this platform can be applied for veterinary medicine settings. Further, it supports the potential for repurposing known anti-trichomonals for veterinary use and vice versa*.* Although we show this assay can be applied in rapid HTS for *T. foetus*, we recommend further optimisation of this assay, specifically lowering the seed number of *T. foetus* to compensate for their rapid growth in the conditions tested.

Several studies have highlighted the potential of natural products as anti-trichomonals (Bala and Chhonker, 2018), particularly from microbial sources (de Brum Vieira et al., 2015; King et al., 2019). We performed the largest pure-compound microbial natural product screen against *T. vaginalis* to date and identified novel anti-trichomonal activities across numerous natural compound classes, emphasising that more frequent and broader screens are required. Most notable among these, we identified fumagillin as a potent anti-trichomonal, and further explored it as a broad-spectrum, anti-protistal compound. *In silico* docking support conserved interactions of fumagillin with protistal MetAP2s, consistent with its known molecular MOA in higher eukaryotes (Griffith et al., 1998). In humans, analogues of fumagillin represent potential anti-cancer agents, including some in clinical trials, where inactivation of MetAP2 inhibits angiogenesis crucial to tumour growth (Griffith et al., 1997). Despite its prior study as a potential cancer treatment, the selective potency of fumagillin against protists is highlighted by its near nanomolar IC_50_ values in protist species reported herein and elsewhere (Arico-Muendel et al., 2009a; Chen et al., 2009, 2011), which should produce a large enough therapeutic window to limit dose-dependent toxicity in the host (i.e. selecting low doses toxic to parasites whilst remaining non-toxic against other cells). Of further note, studies of protistal MetAP2 sequence and predicted structures have highlighted differences between human orthologs in amino acid side chains in the enzyme binding pocket (Zhang et al., 2002), and the potential that these differences can be leveraged towards improved protist specificity in synthesis of similar epoxide analogues. If this selectivity can be maximised, it would complement the absence of anti-bacterial activity we observed for fumagillin, and avoid indirect toxicity against diverse host microbiomes, which has emerged as a particular issue in Mtz treatment of gastrointestinal protists (Beatty et al., 2017; Cotton et al., 2015).

Overall, this study describes a novel, simple and effective anti-trichomonal drug-discovery platform that is cost-effective, scalable and high-throughput. By doing so, we hope to accelerate drug-discovery efforts in this globally prevalent parasite, and drive new incentives in pharmaceutical companies to develop next-generation therapeutics against this neglected parasite, with potential also for novel treatment development in veterinary medicine as well.

## Declaration of competing interest

The authors declare that they have no known competing financial interests or personal relationships that could have appeared to influence the work reported in this paper.

## Appendix A Supplementary data

The Supplementary Information contains the complete supplementary data, including Supplementary Data and their descriptions, Supplementary Figures and their legends. These data will be made available as a document during submission and peer review, but if the paper is accepted these will be made available through Mendeley as stated in Methods 2.6.
