# Supplementary material for: TriTOX: A novel Trichomonas vaginalis assay platform for high-throughput screening of compound libraries
Source: Int J Parasitol Drugs Drug Resist. 2021 Feb 10;15:68–80. doi: 10.1016/j.ijpddr.2021.01.001 (PMC7897990; doi:10.1016/j.ijpddr.2021.01.001)

Supplementary Information

TriTOX: a novel *Trichomonas* sp. assay platform for high-throughput screening of metabolite libraries.

Alexander Y.F. Lam[1,*], Daniel Vuong[2], Aaron R. Jex[1,3,*], Andrew M. Piggott[4], Ernest Lacey[2,4] & Samantha J. Emery-Corbin[1,*],

[1] Population Health and Immunity Division, The Walter and Eliza Hall Institute of Medical Research, Melbourne, VIC, Australia
[2] Microbial Screening Technologies, Smithfield, NSW, Australia
[3] Department of Veterinary Biosciences, Melbourne Veterinary School, Faculty of Veterinary and Agricultural Sciences, The University of Melbourne, Parkville, VIC, Australia
[4] Department of Molecular Sciences, Faculty of Science and Engineering, Macquarie University, North Ryde, NSW, Australia
*Department of Medical Biology, The University of Melbourne, Parkville, VIC, Australia

Corresponding Author:
Dr Samantha Jane Emery-Corbin
Population Health and Immunity Division, The Walter and Eliza Hall Institute of Medical Research
Parkville, Victoria, 3052
Australia
Email: emery.s@wehi.edu.au
Phone: +61-3-9345-2656

Running Title: TriTOX drug-screening platform in *Trichomonas vaginalis*

Keywords: *Trichomonas*, *Tritrichomonas*, Drug-discovery, natural products, microbial metabolites

Supplementary Data:

Supplementary Data 1: Excel spreadsheet documenting all compounds within the microbial metabolite library as well as the % inhibition for *T. vaginalis*. The inhibitory activity of mammalian cell lines NS-1 (anti-tumour) and NFF (cytotoxity) is also shown, with active (A) compounds having >25% inhibitory activity otherwise these were reported as inactive (I).

Supplementary Tables and legends:

Supplementary Table S1: Pre-optimisation of assay conditions for *T. vaginalis* and *T. foetus* growth **(A)**. Mtz IC_{50} values in 96-well plates for anaerobic incubation conditions. **(B)**. Z′ values in 96-well plates for anaerobic incubation conditions

A)

	T. vaginalis			*T. foetus*		
		**100 µL**	**200 µL**		**100 µL**	**200 µL**
**40 hours**	**10 cells/µL**	1.63	1.63	**10 cells/µL**	1.17	1.19
	**20 cells/µL**	2.64	2.16	**20 cells/µL**	1.31	1.23
		**100 µL**	**200 µL**		**100 µL**	**200 µL**
**65 hours**	**10 cells/µL**	3.09	2.67	**10 cells/µL**	2.17	2.07
	**20 cells/µL**	3.21	3.19	**20 cells/µL**	2.70	2.38

B)

	T. vaginalis			*T. foetus*		
		**100 µL**	**200 µL**		**100 µL**	**200 µL**
**40 hours**	**10 cells/µL**	0.418	0.726	**10 cells/µL**	0.357	0.769
	**20 cells/µL**	0.418	0.723	**20 cells/µL**	0.339	0.773
		**100 µL**	**200 µL**		**100 µL**	**200 µL**
**65 hours**	**10 cells/µL**	0.346	0.812	**10 cells/µL**	0.313	0.493
	**20 cells/µL**	0.453	0.544	**20 cells/µL**	0.366	0.540

Supplementary Table S2: Miniaturisation of assay conditions for *T. vaginalis* growth. **(A)**. Mtz IC_{50} values in 96- and 384-well plates for *T. vaginalis* anaerobic incubation conditions. Diagonal lines within cells indicate no IC_{50} value was obtained as no dose-response curve could be fitted. **(B)**. Z′ values in 96- and 384-well plates for *T. vaginalis* anaerobic incubation conditions

A)

	96-well plates			384-well plates		
		**50 µL**	**100 µL**		**25 µL**	**50 µL**
**24 hours**	**15 cells/µL**		2.91	**250 cells/well**	1.22	0.759
	**50 cells/µL**	2.18	2.23	**500 cells/well**	2.02	1.36
				**1000 cells/well**	3.21	2.33
		**50 µL**	**100 µL**		**25 µL**	**50 µL**
**36 hours**	**15 cells/µL**	1.16	1.34	**250 cells/well**	0.623	0.672
	**50 cells/µL**	3.74	3.45	**500 cells/well**	0.888	0.703
				**1000 cells/well**	1.89	0.893
		**50 µL**	**100 µL**		**25 µL**	**50 µL**
**48 hours**	**15 cells/µL**	1.58	1.96	**250 cells/well**	1.55	1.34
	**50 cells/µL**	3.49	5.01	**500 cells/well**	2.90	1.50
				**1000 cells/well**	4.31	3.03

B)

	96-well plates			384-well plates		
		**50 µL**	**100 µL**		**25 µL**	**50 µL**
**24 hours**	**15 cells/µL**	-0.247	-0.873	**250 cells/well**	0.802	0.787
	**50 cells/µL**	0.843	0.666	**500 cells/well**	0.847	0.775
				**1000 cells/well**	0.711	0.863
		**50 µL**	**100 µL**		**25 µL**	**50 µL**
**36 hours**	**15 cells/µL**	0.764	0.506	**250 cells/well**	0.861	0.867
	**50 cells/µL**	0.784	-0.0419	**500 cells/well**	0.931	0.718
				**1000 cells/well**	0.556	-0.443
		**50 µL**	**100 µL**		**25 µL**	**50 µL**
**48 hours**	**15 cells/µL**	0.741	0.889	**250 cells/well**	0.870	0.796
	**50 cells/µL**	0.725	0.849	**500 cells/well**	0.866	0.450
				**1000 cells/well**	0.630	0.828

Supplementary Figure Legends:

Supplementary Figure S1: Changes in pH and absorbance of blank media over time**. (A)**. Change in pH of controls (blank media) over time Data plotted as mean±SD. **(B)**. Change in absorbance of controls (blank media) over time. Data points at 0 hours were measured on a different machine. Data plotted as mean±SD.

Supplementary Figure S2: Dose-response curves for Mtz across pre-optimisation assay conditions for *T. foetus* and *T. vaginalis* trophozoites. Data plotted as mean±SEM, n=4.

Supplementary Figure S3: Dose-response curves for Mtz across miniaturising medium and high-throughput assay conditions for *T. vaginalis* trophozoites. Data plotted as mean±SD.

Supplementary Figure S4: Effect of varying chromogen concentration during assay condition C5 (384-well plates, 25µL/well, 10,000trophozoites/mL, 48 hours of anaerobic incubation) for several anti-trichomonal compounds. **(A)**. Dose-response curves for inhibitory compounds against *T. vaginalis* trophozoites. Data plotted

as mean±SD. **(B)**. Bar plot depicting the differences in absolute absorbance values (arbitrary units) between positive and negative control wells. Data plotted as mean±SD.

Supplementary Figure S5: Representative photos of *T. vaginalis* growth (10,000 cells/mL) in the presence of 5% chromogen in **(A)**. 200 µL wells and **(B)**. 100 µL wells, incubated for 40 hours. Both assay plates were set up as follows: Row A was supplemented with 0.5% DMSO as a vehicle control and Row H contained 'blank' wells (i.e. no *T. vaginalis* trophozoites). Rows B to G shows *T. vaginalis* growth in the presence of 100 µg/mL (column 1) Mtz, tinidazole, furazolidone, albendazole, quinacrine and paromomycin respectively, serially diluted 2-fold across the row.

Supplementary Figure S6: Chemical structures of identified anti-trichomonal compounds and their structural analogues present in the microbial metabolite library

Supplementary Figure S7: Dose-response curves for microbial metabolites against *T. vaginalis* and *T. foetus* trophozoites. Data plotted as mean±SD.

Supplementary Figure S8: Multiple sequence alignment of human (P50579_MetAP2_Human) and orthologous protistal MetAP2 (TVAG_476160 for *T. vaginalis*, GL50803_86600 for *G. lamblia* and EHI_126880 for *E. histolytica*).

Conserved histidine (His) at position 231 of human MetAP2 implicated for covalent bond formation with Fumagillin is indicated with red arrow, as well as equivalent residues in protists (His164 for *T. vaginalis*, His165 for *G. lamblia* and *E. histolytica*). Residues are coloured according to colour pattern in ALSCRIPT Calcons (Aline), indicating red=identical residues, while a scale of orange to cyan indicates (from most to least) conservation of amino acids' chemical properties at each residue.

Supplementary Figure S1

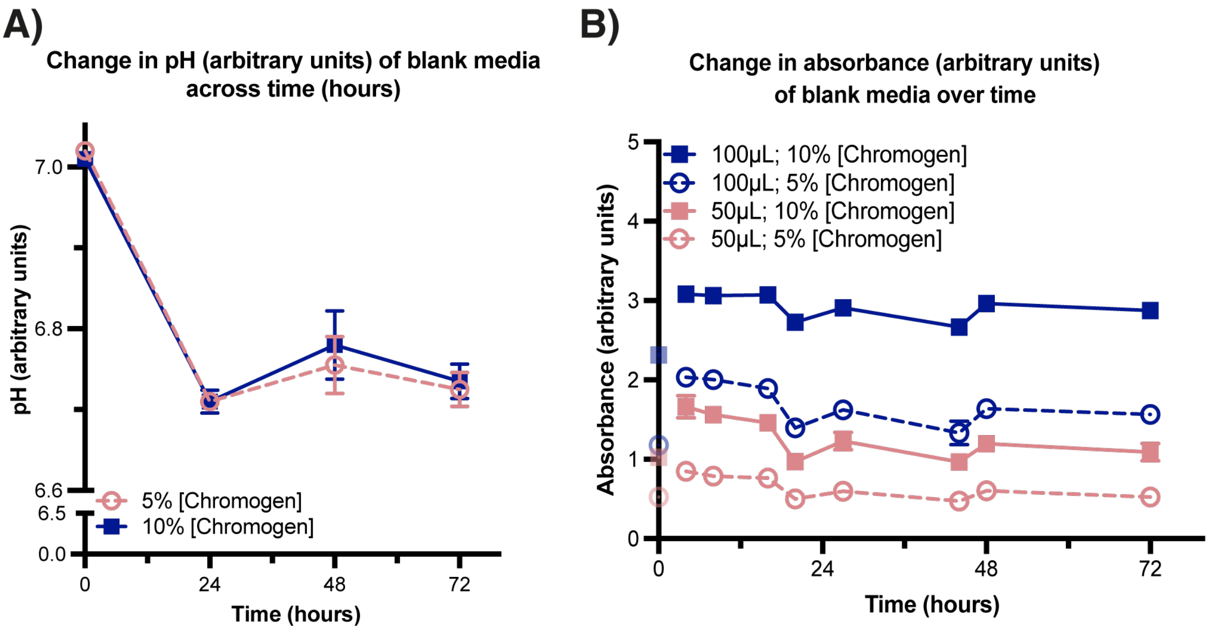

Supplementary Figure S2

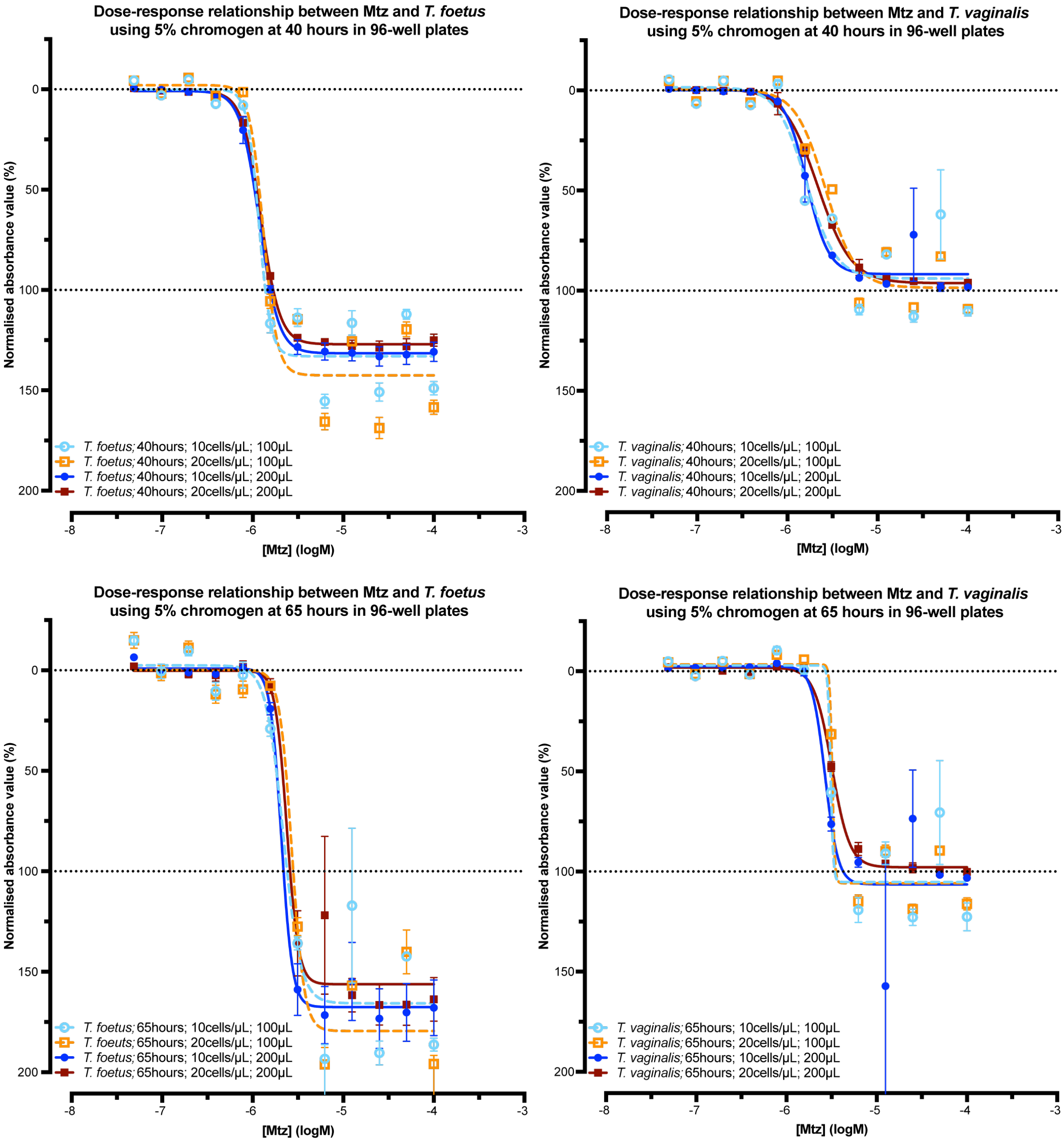

Supplementary Figure S3

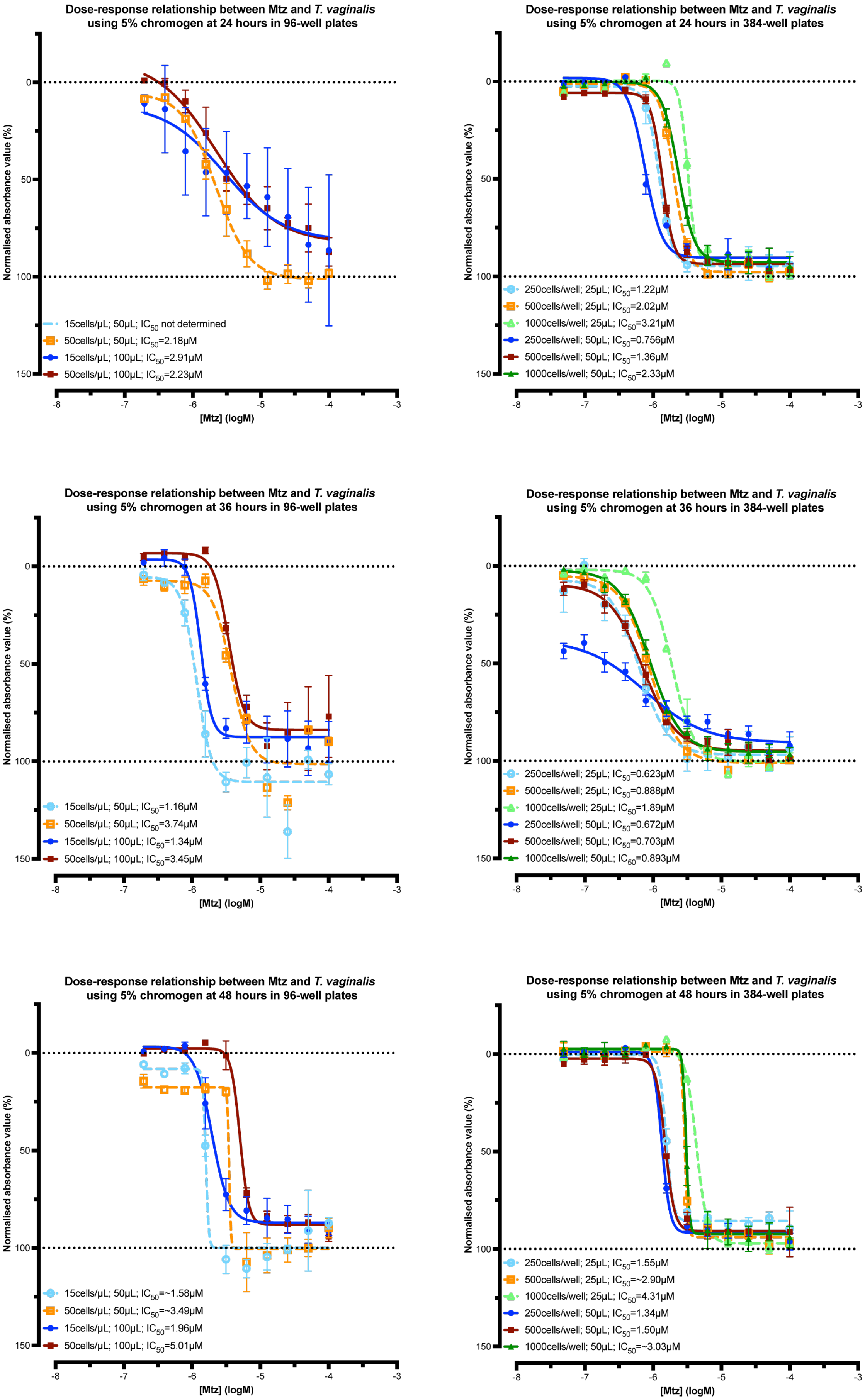

Supplementary Figure S4

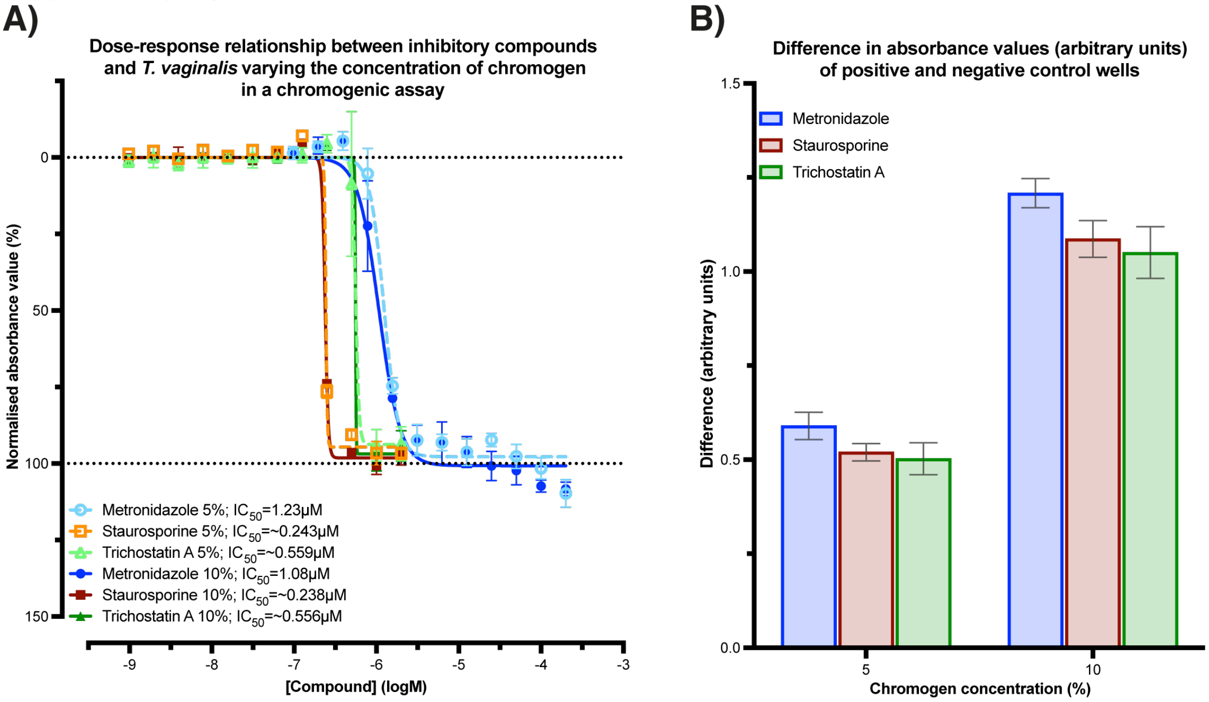

Supplementary Figure S5

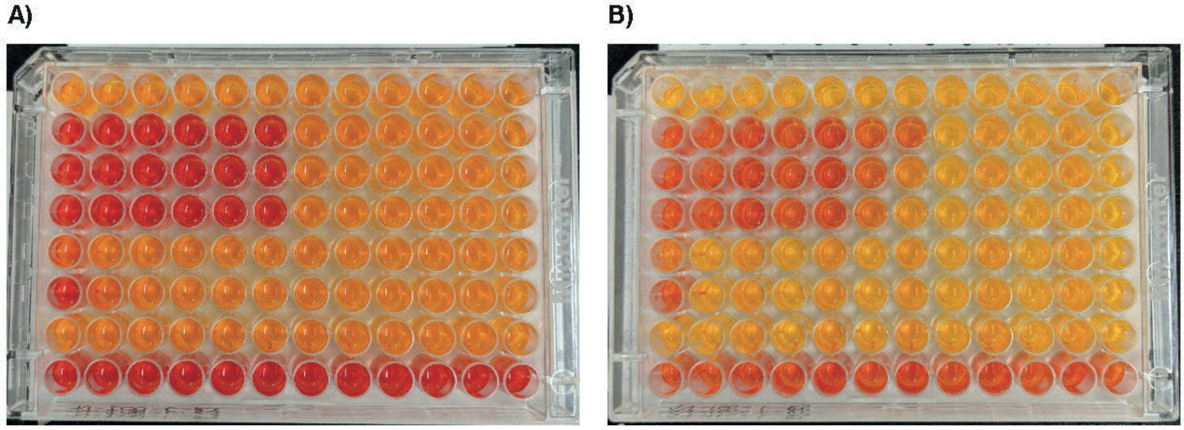

Supplementary Figure S6

NUCLEOTIDES AND NUCLEOBASES

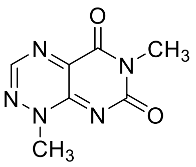

Toxoflavin

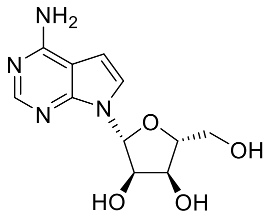

Tubercidin

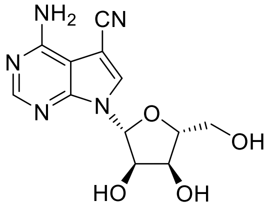

Toyocamycin

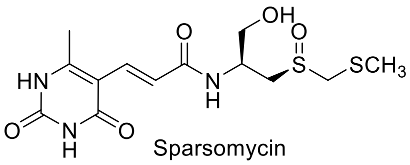

Sparsomycin

POLYENE

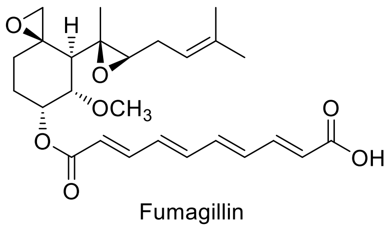

Fumagillin

DITHIOLOPYRROLONES

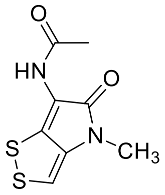

Thiolutin

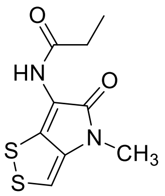

Aureothricin

INDOLOCARBAZOLES

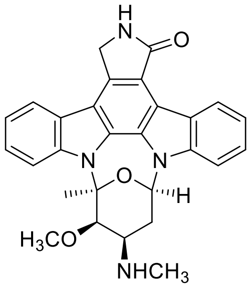

Staurosporine

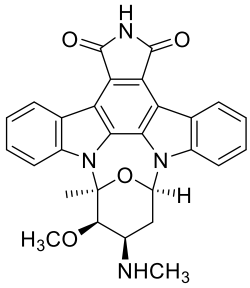

7-Oxostaurosporine

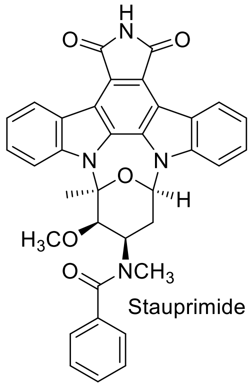

Stauprimide

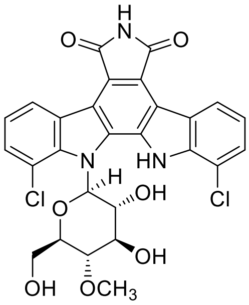

Rebeccamycin

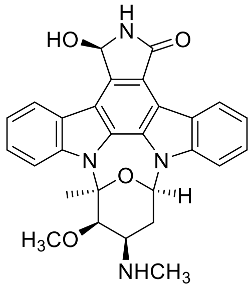

UCN-01

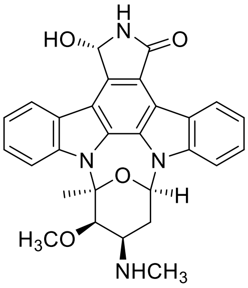

UCN-02

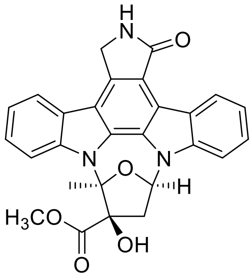

K252A

XANTHONES

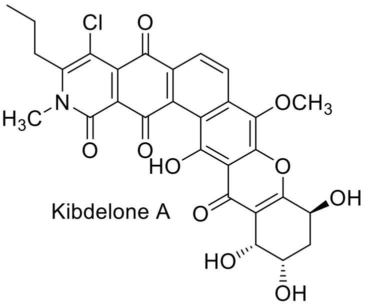

Kibdelone A

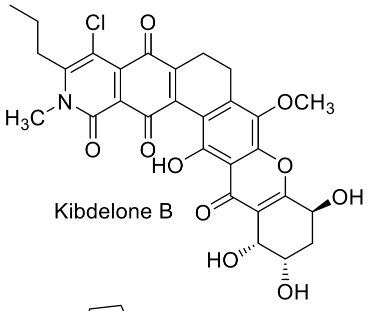

Kibdelone B

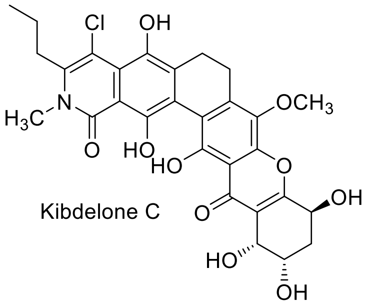

Kibdelone C

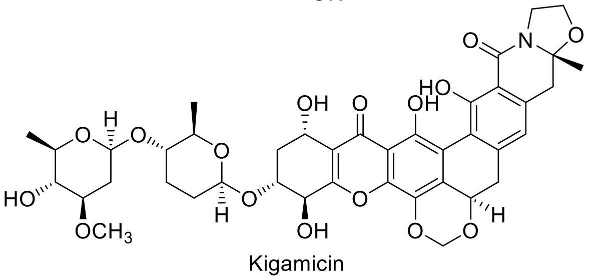

Kigamicin

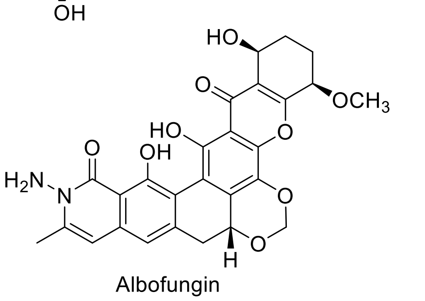

Albofungin

MACROCYCLIC LACTONES

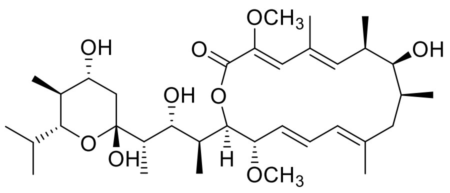

Bafilomycin A1

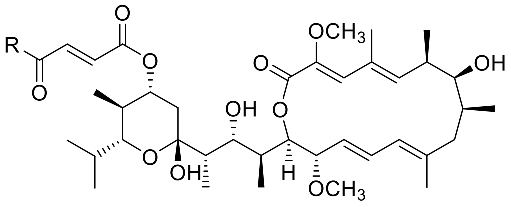

Bafilomycin B1 R = 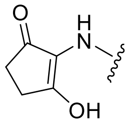

Bafilomycin C1 R = OH

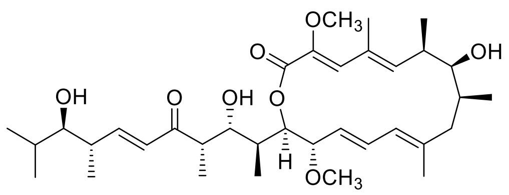

Bafilomycin D

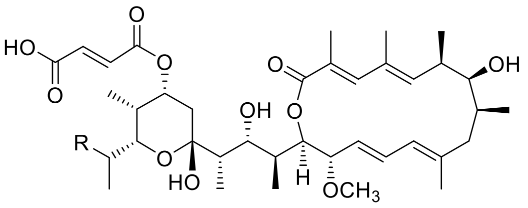

Hygrolidin R = H

TS 155-2 R = CH_3

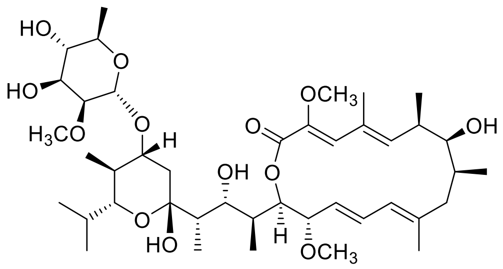

Leucanicidin

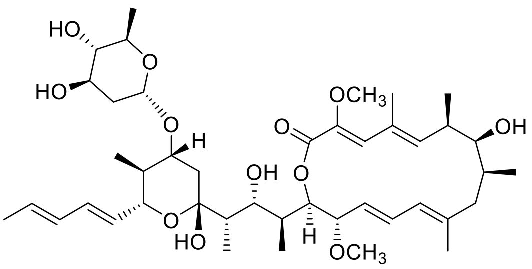

PC-766B

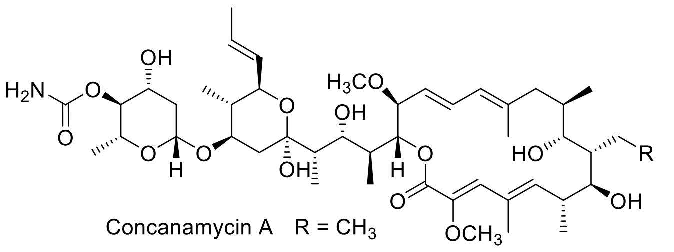

Concanamycin A R = CH_3

Concanamycin B R = H

Supplementary Figure S7

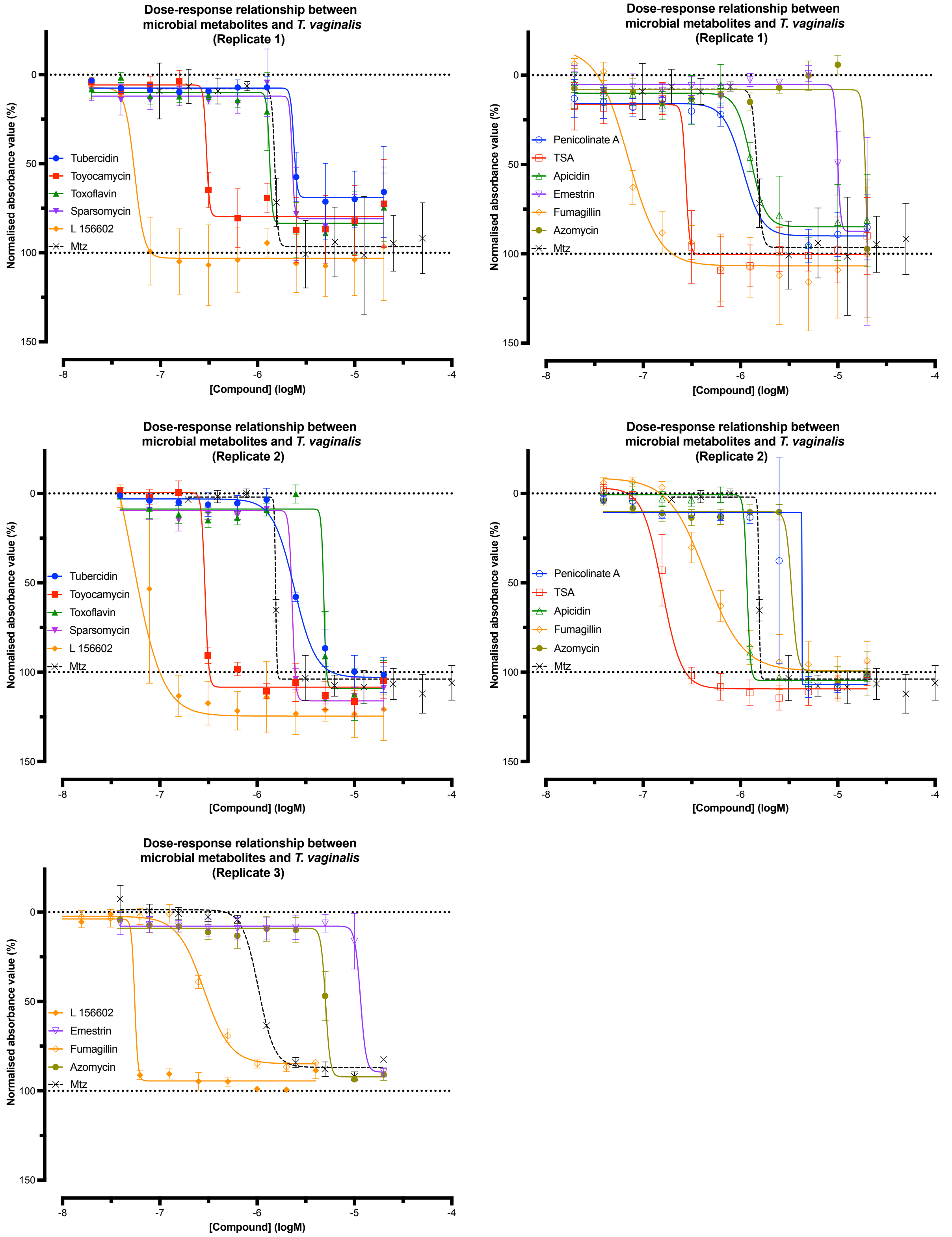

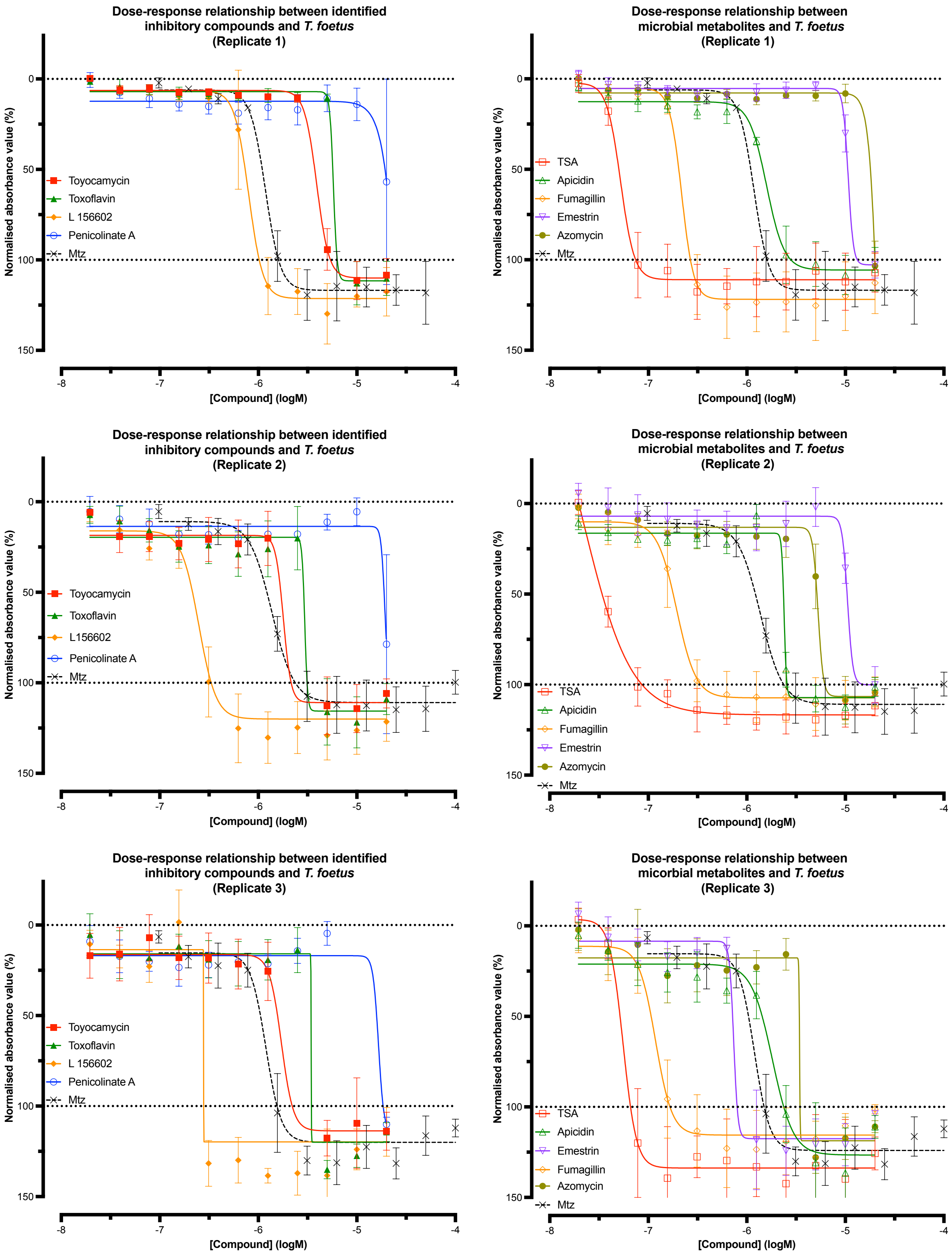
Dose-response relationship between identified inhibitory compounds and T. foetus (Replicate 1)
Normalised absorbance value (%)
Toyocamycin
Toxoflavin
L 156602
Penicolinate A
Mtz
[Compound] (logM)
Dose-response relationship between microbial metabolites and T. foetus (Replicate 1)
TSA
Apicidin
Fumagillin
Emestrin
Azomycin
Mtz
Dose-response relationship between identified inhibitory compounds and T. foetus (Replicate 2)
Toyocamycin
Toxoflavin
L156602
Penicolinate A
Mtz
Dose-response relationship between microbial metabolites and T. foetus (Replicate 2)
TSA
Apicidin
Fumagillin
Emestrin
Azomycin
Mtz
Dose-response relationship between identified inhibitory compounds and T. foetus (Replicate 3)
Toyocamycin
Toxoflavin
L 156602
Penicolinate A
Mtz
Dose-response relationship between micorbial metabolites and T. foetus (Replicate 3)
TSA
Apicidin
Emestrin
Fumagillin
Azomycin
Mtz

Supplementary Figure S8

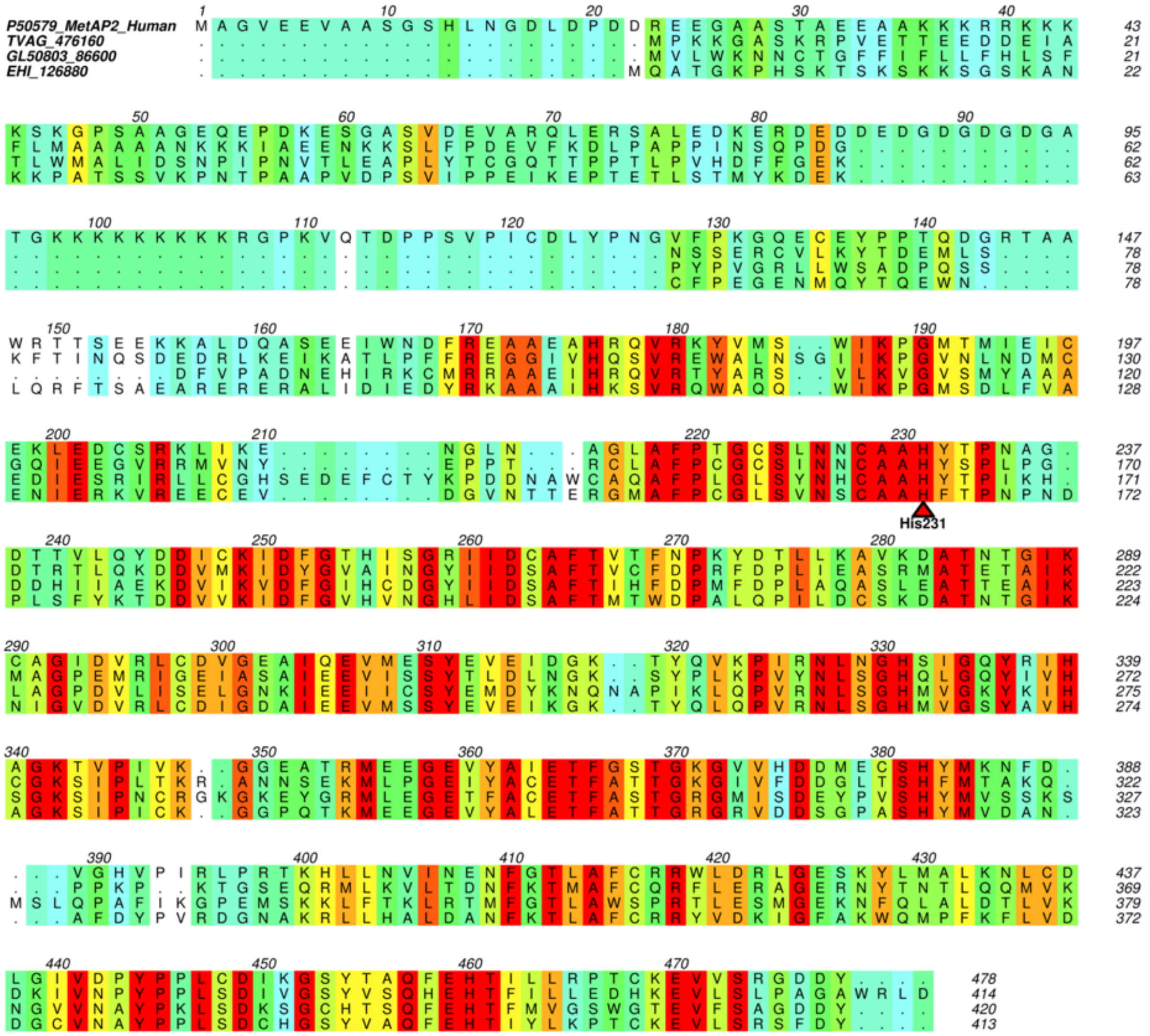

Supplement: Multimedia component 2 [file mmc2.pdf]
